# CD14 and Complement Crosstalk and Largely Mediate the Transcriptional Response to *Escherichia coli* in Human Whole Blood as Revealed by DNA Microarray

**DOI:** 10.1371/journal.pone.0117261

**Published:** 2015-02-23

**Authors:** Corinna Lau, Ståle Nygård, Hilde Fure, Ole Kristoffer Olstad, Marit Holden, Knut Tore Lappegård, Ole-Lars Brekke, Terje Espevik, Eivind Hovig, Tom Eirik Mollnes

**Affiliations:** 1 Research Laboratory and Department of Laboratory Medicine, Nordland Hospital, Bodø, Norway; 2 Department of Informatics, University of Oslo, Oslo, Norway; 3 Bioinformatics Core Facility and Institute for Medical Informatics, Oslo University Hospital, Oslo, Norway; 4 Department of Medical Biochemistry, OUS, Ullevaal, Oslo, Norway; 5 Norwegian Computing Center, Oslo, Norway; 6 Faculty of Health Sciences, University of Tromsø, Tromsø, Norway; 7 Division of Medicine, Nordland Hospital, Bodø, Norway; 8 Center of Molecular Inflammation Research, Department of Cancer Research and Molecular Medicine, Norwegian University of Science and Technology, Trondheim, Norway; 9 Department of Tumor Biology, Institute for Cancer Research, Oslo University Hospital, Oslo, Norway; 10 Department of Cancer Genetics and Informatics, Oslo University Hospital, Oslo, Norway; 11 Institute of Immunology, Oslo University Hospital Rikshospitalet and University of Oslo, Oslo, Norway; INSERM, FRANCE

## Abstract

Systemic inflammation like in sepsis is still lacking specific diagnostic markers and effective therapeutics. The first line of defense against intruding pathogens and endogenous damage signals is pattern recognition by e.g., complement and Toll-like receptors (TLR). Combined inhibition of a key complement component (C3 and C5) and TLR-co-receptor CD14 has been shown to attenuate certain systemic inflammatory responses. Using DNA microarray and gene annotation analyses, we aimed to decipher the effect of combined inhibition of C3 and CD14 on the transcriptional response to bacterial challenge in human whole blood. Importantly, combined inhibition reversed the transcriptional changes of 70% of the 2335 genes which significantly responded to heat-inactivated *Escherichia coli* by on average 80%. Single inhibition was less efficient (p<0.001) but revealed a suppressive effect of C3 on 21% of the responding genes which was partially counteracted by CD14. Furthermore, CD14 dependency of the *Escherichia coli*-induced response was increased in C5-deficient compared to C5-sufficient blood. The observed crucial distinct and synergistic roles for complement and CD14 on the transcriptional level correspond to their broad impact on the inflammatory response in human blood, and their combined inhibition may become inevitable in the early treatment of acute systemic inflammation.

## Introduction

Systemic inflammatory conditions are major health problems. For sepsis, which has a lethality rate of 20% to 60%, we lack both effective therapeutics and specific diagnostic markers. Clinical studies of potential therapeutics have largely failed, possibly due to (i) the use of single interventions, (ii) lack of patient stratification or (iii) inappropriate timing [1,2]. We hypothesize that upstream targeting of the innate immune response by combined inhibition of complement and Toll-like receptor (TLR) signaling at the levels of complement factors C3 or C5 and TLR co-receptor CD14, respectively, may constitute a suitable therapeutic strategy for broad and early treatment of acute systemic inflammation [3,4].

Systemic inflammation can be induced by a broad variety of exogenous and endogenous danger signals represented by pathogen-associated molecular patterns (PAMPs), such as bacterial toxins and structural components, fungi, and viral nucleic acids, as well as damage-associated molecular patterns (DAMPs), such as necrotic cells and endocrine glycolipids [5]. PAMPs and DAMPs are recognized by pattern recognition receptors (PRRs) of the host innate immune system, including TLRs and the complement system. CD14 is a key molecule in TLR signaling and C3 and C5 are key molecules of the complement system. Together, they represent potential candidates for therapeutic targeting [3].

Dysregulation of complement is involved in a plethora of diseases. Three different complement pathways converge at the step of C3 activation by cleavage to C3a and C3b. C3a is an anaphylatoxin that signals through C3aR, while the C3b inactivation product iC3b is involved in complement-mediated opsonization of microbial and particle surfaces and their subsequent phagocytosis [6]. Further, C3b becomes part of the C5 convertase, which activates C5 by cleavage into C5a and C5b. C5a signals through its two receptors, C5aR and C5L2. C5b is involved in the formation of the terminal complement complex (TCC), which as lipid membrane associated form (membrane attack complex) might lyse Gram-negative bacteria like *Escherichia coli* (*E*. *coli*), or host cells. In sub-lytic doses it activates host cells to release inflammatory mediators. C5 can also be activated in the absence of C3, upon cleavage by proteases from the coagulation cascade, e.g. thrombin and proteases from phagocytic cells [7].

CD14 signals through interactions with different Toll-like receptors (TLRs) upon binding of acylated structural components e.g., derived from Gram-positive and Gram-negative bacteria [8,9]. Except TLR3, all TLRs signal through the adaptor molecule Myeloid Differentiation Factor 88 (MyD88)-dependent pathway, which is initiated at the plasma membrane and rapidly activates transcription factors NF-κB and AP1. Additionally, encounter of TLR3 and TLR4 initiates a MyD88-independent, TIR-domain-containing adapter-inducing interferon-β (TRIF)-dependent pathway, which occurs at early endosomes and activates interferon regulatory factor-3 (IRF3) and NF-κB [10].

CD14 and TLR activation leads to both innate and adaptive immune responses [11] and to the expression of a wide variety of pro- and anti-inflammatory cytokines and chemokines. Through binding to specific cell surface receptors on specific target cells, cytokines and chemokines mediate among others chemotaxis, vascular cell adhesion, cytotoxicity, and cellular proliferation and differentiation. TLR signaling can be modulated by a variety of intracellular signal transducers, which have also been proposed to mediate signaling by complement receptors, such as β-arrestins and the anti-inflammatory G-protein alpha subunit Gαi2 [12–14]. Also, subsequent activation of mitogen-activated protein kinase (MAPK) ERK1/2 and NF-κB are common events downstream of both TLR and complement receptor engagement. The encounter of these mutual second messengers contributes to crosstalk between complement and CD14/TLR signaling [15].

We have earlier demonstrated that CD14 and complement play cell type-specific roles on the level of cytokine and chemokine responses, granulocyte enzyme release and oxidative burst in a model of Gram-negative bacteria-induced inflammation using whole blood from healthy donors and a C5-deficient patient [16]. The present study aimed to characterize the *E*. *coli*-induced inflammatory response in human whole blood on the transcriptional level and revealed a large potential of combined inhibition of CD14 and complement to neutralize this transcriptional response.

## Results

### *Escherichia coli* responsive genes in human whole blood

Microarray technology was applied to decipher the transcriptional response to inflammatory stimuli contained by heat-inactivated *E*. *coli* in fresh human whole blood samples. The expression level of 2335 (12%) of in total 19,695 detectable transcripts changed significantly upon bacterial challenge. These transcripts were defined as *E*. *coli*-responsive genes (*ERG*s) and applied to further statistical and functional annotation analyses. Of all *ERG*s, 1097 (47%) were up-regulated, 1238 (53%) were down-regulated, and 362 (16%) responded with transcriptional changes of more than two-fold (Table 1). Supporting qPCR experiments were performed (see *Validity of microarray data* below).

**Table 1:** *E*. *coli*-responsive genes (*ERG*s) and their sensitivity to single or combined inhibition of CD14 and C3.

Category	Number of transcripts^A^		
	Total	Reversible	Augmentable
***ERG*s**	2335^B^	1892^C^	105^D^
*Up-regulated*	1097	870	81
*Down-regulated*	1238	1022	24
*FC > 2* ^E^	362	338	11
**C3/CD14-DG** ^F^	1687	1626	61
**CD14-DG** ^G^	1339	1323	16
**C3-DG** ^H^	827	334	493

^A^ Affected transcripts may count redundantly in different categories; See Supplementary tables for data for inhibition of C5a receptor (S2 Table) and for data from a C5-deficient patient (S1 Table)

^B^ Of 2335 *ERG*s, 338 were not found among neither the reversible (n = 1892) nor augmentable (n = 105).

^C^ Reversed by at least one inhibitory strategy

^D^ Not reversible at all

^E^ Fold change (FC) expression in response to *E*. *coli* above two-fold

^F^ C3- and CD14-dependent genes (sensitive to combined inhibition of C3 and CD14)

^G^ CD14-dependent genes (sensitive to inhibition of CD14 with anti-CD14)

^H^ C3-dependent genes (sensitive to inhibition of C3 with compstatin)

### Effects of CD14 and C3 inhibition on *E*. *coli*-induced gene expression

**Fig 1:**
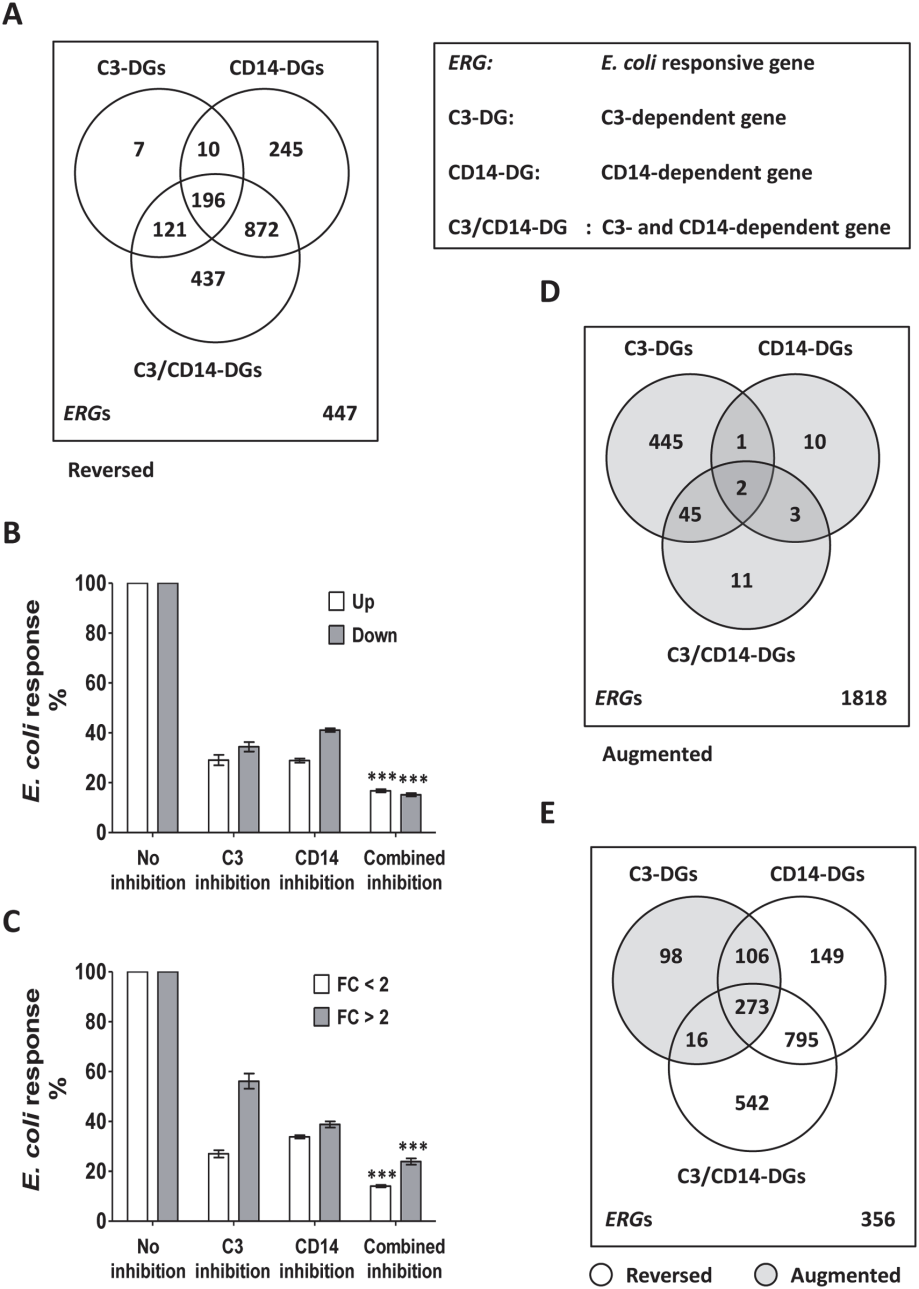
CD14 and/or C3 inhibition of the transcriptional response to *E*. *coli*. A, The diagram shows common and specific groups of reversible C3-dependent genes (C3-DGs), CD14-DGs and C3/CD14-DGs DGs. Common genes are encompassed by more than one circle. The sum of all numbers within the diagrams equals the total number of *ERG*s (n = 2335). Numbers of *ERG*s which belong to none of the respective DG groups are indicated at the bottom right of the diagram. B and C, The remaining *E*. *coli*-induced transcriptional responses of reversible *ERG*s (in % of total) in the presence of inhibitors were derived from the transcriptional response in the presence of inhibitors of C3 (n = 334), CD14 (n = 1323) or both (combined inhibition; n = 1626) divided by the uninhibited response (set to 100%). Data are shown for up- and down-regulated *ERG*s (B), and for *ERG*s with fold change (FC) responses above (FC<2) or below two-fold (FC<2) (C). Data are given as mean and SEM. The significances of the differences between combined inhibitory effect and single inhibitory effects were determined by Two-way ANOVA and Bonferroni post-testing (***, *p*<0.001). D and E, The Venn diagrams show augmentable C3-DGs, CD14-DGs and C3/CD14-DGs (D) or augmentable C3-DGs (gray circles) compared to reversible CD14-DGs and C3/CD14-DGs (white circles) (E). The total numbers of DGs are listed in Table 1.

Combined inhibition of CD14 and complement at the level of C3 was the most effective inhibitory strategy and reversed the transcriptional response of 70% (n = 1626) of all *ERG*s (reversible C3/CD14-dependent genes (C3/CD14-DGs)) (Table 1). Combined inhibition was even necessary in order to reverse the *E*. *coli* response of 437 *ERG*s, which were not affected by single inhibition of CD14 or C3 (Fig. 1A). For the remaining reversible C3/CD14-DGs (n = 1189), CD14 played a more prominent role than complement and mediated the response of 66% (n = 196+872) of those genes compared to 19% (n = 196+121) for C3-inhibition (Fig. 1A). In addition, 98% (n = 196+121+10) of the reversible C3-DGs were also sensitive to single inhibition of CD14 or combined inhibition (Fig. 1A). Finally, 15% (n = 338) of all *ERG*s were not sensitive to inhibition of CD14 and/or complement at all (Table 1, 2^nd^ footnote) and were, thus, assumed to be independent of CD14 and C3. See S1 Table and S1 Fig. for respective data on *E*. *coli* responsiveness and inhibitory effects in C5-deficient blood with or without reconstitution with recombinant C5.

We also tested the effect of a C5a receptor antagonist on the *E*. *coli* response (S2 Table) in order to decipher differential C3- and C5aR-dependencies. In accordance with its upstream position, we found dominant roles for C3 in the response of C3-DGs, C5aR-DGs (S2 Fig., panels A and B) and C5-dependent genes (genes that were significantly different regulated in response to *E*. *coli* in C5-deficient blood compared to normal blood) (S2 Fig., panel C).

### Degree of reversion of the *E*. *coli* response in the presence of C3 and/or CD14 inhibitors

Inhibitory effects of single and combined inhibition on the *E*. *coli* response were estimated as ratio between gene expression levels in presence compared to absence of inhibitor for reversible DGs. Combined inhibition of C3 and CD14 reversed *E*. *coli*-induced transcriptional changes more potently (*p*<0.001) than single inhibitions, and reduced the *E*. *coli* responses of both, up- and down-regulated *ERG*s by more than 80% (Fig. 1B). Expression changes above two-fold were slightly less efficiently reduced than changes below two-fold, but with the same significant differences (Fig. 1C).

### Suppressive effect of C3 on *E*. *coli* response counteracted by CD14

Upon inhibition of CD14 or both, CD14 and C3, most of the *E*. *coli* responses could be reversed (Table 1). In contrast, inhibition of C3 alone increased the responses of 60% of the C3-DGs (n = 493), which is 21% of all *ERG*s. Thus, C3 appeared to have a substantial suppressive effect on the *E*. *coli*–induced transcriptional response in human blood. A similar effect was found for inhibition of the C5aR (CD88) (S2 Table), whereby the majority of C5aR-DGs were also C3-DGs (S2 Fig., panel B). Notably, 90% (n = 445) of the augmented C3-DGs were solely augmented upon C3 inhibition (Fig. 1D), and 77% (n = 379) of them were CD14-dependently reversed either upon single CD14-inhibition (n = 106) or combined inhibition (n = 273) (Fig. 1E). Thus, the putative suppressive function of C3 may largely be counteracted by CD14. In a C5-deficient background (S1 Table), the proportion of augmentable *ERG*s (0.1%) was heavily decreased compared to healthy individuals (4.5%) (Table 1).

### Validation of microarray data

**Fig 2:**
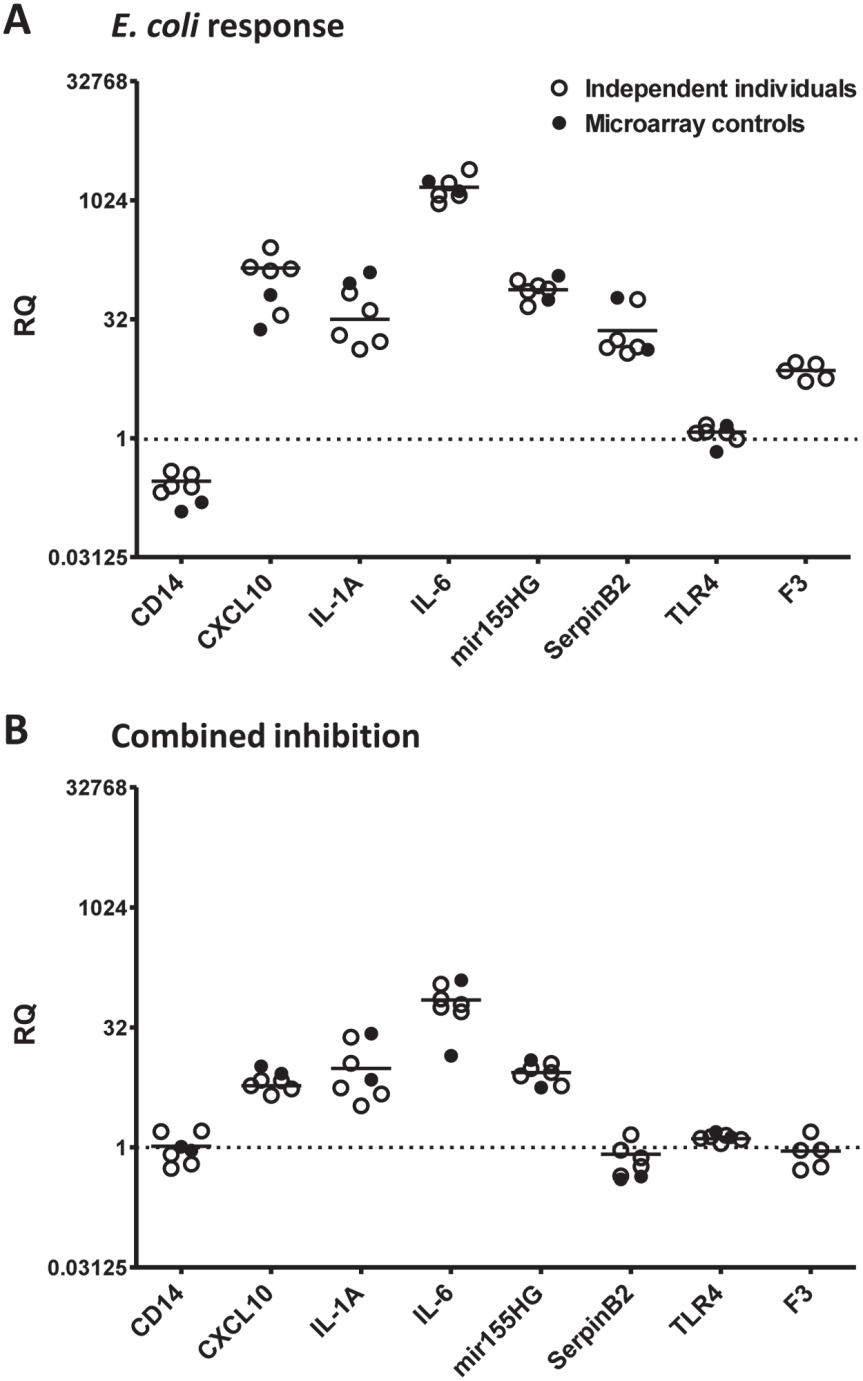
Inter-individual variations of the *E*. *coli* response of selected genes. Relative quantification (RQ) data from qPCR analyses are displayed as scatter plots for the *E*. *coli* response of seven *ERG*s and non-responding TLR4 in absence (A) and presence (B) of combined inhibition of C3 and CD14. Data are shown for two sets of data, the controls included in the microarray study (●; single data points, n = 2) and five independent individuals (○; single data points, n = 5 with mean). The expression data were normalized to spontaneous activation in presence of PBS, only, which is indicated as a dotted line crossing the Y-axis (log_2_ scale) at RQ = 1. No statistical significance of the differences between the two datasets was found for the uninhibited (P = 0.82) and the inhibited response (P = 0.89) in a two-tailed unpaired t-test with Welch’s correction.

qPCR experiments on five independent healthy individuals were performed to validate the microarray data, which were based on only two control individuals. As expected, we observed reasonable inter-individual variation of the transcriptional response to *E*. *coli* for all tested genes (Fig. 2). However, the responses were of similar patterns among all individuals. There was no significant difference between the qPCR expression data from the five independent individuals and the two control individuals used for the microarray study (*p*>0.05). The two datasets correlated significantly with R^2^ = 0.99 (*p*<0.0001) for both the uninhibited (Fig. 2A) and the inhibited response (Fig. 2B).

According to the microarray data, also overall differential gene expression between the two control individuals did not differ significantly, showing FDR *q*-values above 5% for more than 95% of all *ERG*s for *E*. *coli* response and combined inhibition and for more than 99% for single inhibitions. Notably, the microarray data of the two control individuals were also technically verified by qPCR (S6 Fig.), and relative quantification equivalents of the microarray data correlated perfectly with the qPCR data (R^2^ = 0.96, *p*<0.0001).

Despite the observed inter-individual variation of the uninhibited *E*. *coli* response (Fig. 2A), we found a substantial inhibitory effect of combined inhibition on the *E*. *coli*-induced transcriptional response of all tested *ERG*s (Fig. 2B). The remaining *E*. *coli* response of these *ERG*s in presence of combined inhibition of C3 and CD14, given in % uninhibited response (mean±SEM), was 33.5±7.7 for CD14, 6.3±2.6 for CXCL10, 29.9±4.2 for IL-1A, 5.2±0.8 for IL-6, 11.9±1.5 for miR155, 4.6±1.1 for SerpinB2 and 12.5±2.1 for F3. These results were in agreement with the overall average of 15.9% ± 0.5% for all *ERG*s revealed by microarray analyses (Fig. 1B and C).

### Crosstalk between CD14- and C3-mediated responses

**Fig 3:**
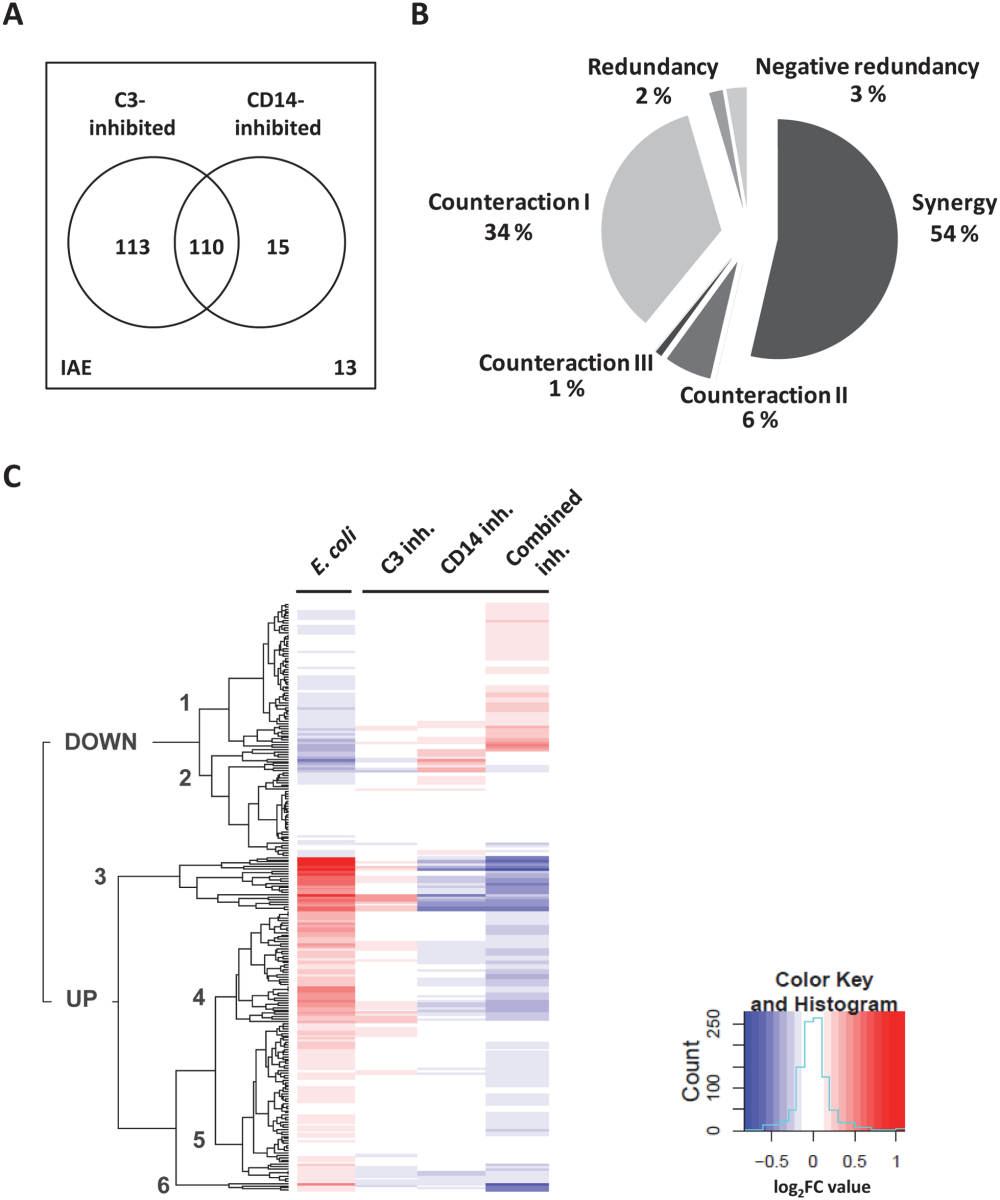
Crosstalk between CD14- and C3-dependent responses. Crosstalk between CD14 and C3 was assumed when the sum of single inhibitory effects was significantly different from the inhibitory effect of combined inhibition in ANOVA-based interaction effect analyses. This scenario was found for 251 *ERG*s, designated IAEs. A, The Venn diagram shows the distribution of *ERG*s with significant effects (ANOVA-based analysis) of single CD14 or C3 inhibition among the 251 IAE genes. Common genes are encompassed by more than one circle. The total numbers for IAEs with significant effects of C3- or CD14-inhibition were 223 and 125, respectively. IAE genes with both single and combined inhibitory effects (n = 110) were designated IAE-I. Numbers of IAEs without single inhibitory effects are indicated at the bottom right of the diagram (n = 13). B, The graph displays the distribution of IAE-I genes among six classes of crosstalk between CD14 and C3 including synergy, four types of counteraction and redundancy. The different crosstalk types were defined according to individual profiles of reversion and augmentation of the *E*. *coli* response upon single and combined inhibition using Limma-derived expression data. C, Hierarchical cluster analysis was based on Limma-derived log_2_FC expression values for the *E*. *coli* response of the 251 IAE genes in the absence (*E*. *coli*) and presence of the inhibitors of C3, CD14 or both. Each line contains the fold change (FC) expression data (log_2_FC) for a single *ERG*. Genes with similar responses are clustered according to the hierarchy indicated to the left. Manual examination of the heat map revealed six main clusters of related either down- (clusters 1 and 2) or up-regulated *ERG*s (clusters 3 to 6). The color key and histogram panel indicate total numbers of transcripts contained by the whole gene expression dataset as a function of their log_2_FC expression values. Negative values are displayed in blue, positive values are displayed in red.

To further decipher the contributions of CD14 and C3 to the transcriptional response to *E*. *coli*, we tested for crosstalk between these two key components of innate immunity with ANOVA (Fig. 3). Crosstalk was assumed if the sum of single inhibitory effects on the *E*. *coli* response of any *ERG* was significantly different (*p*<0.05) from the effect of combined inhibition of CD14 and C3. Such significant interaction effects (IAE) were observed for 11% (n = 251) of all *ERG*s (Fig. 3A; S3 Table).

Of the 251 IAE genes, 110 genes were affected by both single inhibitions (Fig. 3A) and were defined as IAE-I (S3 Table). IAE-I genes could be classified into three types of crosstalk: synergy, counteraction and redundancy. Synergistic regulation by CD14 and C3 was assumed when the combined inhibitory effect was more pronounced than the sum of effects of single inhibitions, which was observed for 59 (54%) of the IAE-I genes (Fig. 3B). For another 38 genes (34%) the suppressive function of C3 (augmented *E*. *coli* response when C3 was inhibited) was lost upon combined inhibition and, thus, likely counteracted by a mediating function of CD14 (counteraction I) (Fig. 3). Further, we observed C3-counteracted CD14-mediated (counteraction II) and C3-counteracted CD14-suppressed (counteraction III) responses, as well as redundancies between C3 and CD14 effects, albeit for a very limited number of genes (Fig. 3B and S3 Table). A similar distribution of crosstalk types was seen upon C5-deficiency (S3 Fig.).

### Molecular processes associated with the *E*. *coli* response in human whole blood

The collectivity of *ERG*s primarily associated with pattern recognition as well as T and B cell signaling (Table 2). *E*. *coli* responses above two-fold change were mostly associated with pathways like cytokine-cytokine receptor interaction and Toll-like receptor signaling as well as transcriptional regulation by cytokine-sensitive STATs, NF-κB and interferon-regulatory factors IRFs (S4 Table).

**Table 2:** Pathway analysis for *ERG*s in human whole blood using IPA.

Category	Top Canonical Pathways	*p*
***ERGs***	Altered T cell and B cell signaling in Rheumatoid arthritis	4.38E-15
	Role of pattern recognition receptors in recognition of bacteria and viruses	9.13E-15
	TREM1 signaling	9.20E-14
*Up-regulated ERGs*	Activation of IRF by cytosolic Pattern Recognition Receptors	4.13E-18
	Altered T Cell and B cell signaling in Rheumatoid arthritis	3.31E-15
	Role of Pattern Recognition Receptors in recognition of bacteria and viruses	1.39E-14
*Down-regulated ERGs*	Fcγ Receptor-mediated phagocytosis in macrophages and monocytes	2.02E-07
	Leukocyte extravasation signaling	2.68E-06
	TREM1 signaling	1.09E-04

The responses of nearly all top ten up- and down-regulated *ERG*s were most efficiently reversed by combined inhibition and to a large extent by inhibition of CD14 (S5 Table and S6 Table). Only, thrombomodulin (*THBD*) was unaffected by combined inhibition despite effects of single inhibitions of CD14 and C3 (S6 Table). The top ten up-regulated *ERG*s (S5 Table) were mainly soluble factors like cytokines and chemokines involved in T- and B-cell activation, acute-phase response, chemotaxis, as well as macrophage- and interferon-mediated inflammatory responses: *IL-6*, *IRG1*, *IL-12B*, *CCL20*, *IL-1A*, *CXCL10*, *IFIT1–3*. Also, the host gene of microRNA miR155, a central regulator of innate and adaptive immune responses upon TLR activation [17], was highly up-regulated. The top ten down-regulated *ERG*s (S6 Table) encoded membrane bound or intracellular signal transducers involved in PRR signaling and coagulation (*CD14*, *FOS*, *NLRP12*, *THBD*), cell proliferation, differentiation and adhesion (*CSFR1*, *miR223* and *VCAN*) and phagocytosis (*MERTK*, *CD163*). For expression data of the top regulated *ERG*s in C5-deficient blood, see S7 Table and S8 Table.

### Gene annotation analyses (DAVID) on inhibition-sensitive *ERG*s

CD14 and/or C3-mediated *E*. *coli*–induced transcriptional responses in human whole blood, represented by reversible *ERG*s (n = 1892), were primarily associated with cytokine-cytokine receptor interactions, chemokine signaling and Toll-like receptor signaling as well as transcriptional regulation by AP1 (S4 Table). Further, common and exclusive subsets of C3-DGs, CD14-DGs and C3/CD14-DGs (Fig. 1) were analyzed for their functional annotation (Table 3). Combined inhibition was found to be essential to significantly reverse responses affecting FcγR-mediated phagocytosis and glucose metabolism (reversible C3/CD14-DGs; n = 437) (Table 3). Reversible C3-DGs (n = 334) and CD14-DGs (n = 1323) were uniquely associated with NF-κB-regulated chemokine signaling and IRF2-regulated TLR and NLR signaling, respectively, while both were associated with STAT-mediated cytokine signaling (Table 3).

**Table 3:** Gene annotation enrichment analysis of specific subsets of C3- and/or CD14-dependent *ERGs* (DAVID^A^; *p*<0.05).

**Category /** *Subcategory*	**n** ^B^	**Molecular pathway (KEGG)**	**Transcription factor**
**C3/CD14-DG** ^C^	1687	Cytokine-cytokine receptor interaction, Chemokine signaling pathway, Toll-like receptor signaling pathway	AML1, IRF2, AP1
*Reversible*	1626	Chemokine signaling pathway, Fc gamma R-mediated phagocytosis, Glycolysis / Gluconeogenesis	IRF2, AP1, AML1
*Reversible C3/CD14-dependent*	437	Fc gamma R-mediated phagocytosis, Chemokine signaling pathway, Glycolysis / Gluconeogenesis	ELK1, GRE, BACH1
*Reversible C3-augmentable*	289	Cytosolic DNA-sensing pathway, RIG-I-like receptor signaling pathway, Toll-like receptor signaling pathway	IRF1, IRF2, IRF7
**CD14-DG** ^D^	1339	Cytokine-cytokine receptor interaction, Toll-like receptor signaling pathway, NOD-like receptor signaling pathway	IRF2, STAT, BACH2
*Reversible*	1323	Cytokine-cytokine receptor interaction, Toll-like receptor signaling pathway, NOD-like receptor signaling pathway	IRF2, STAT, BACH2
**C3-DG** ^E^	827	Toll-like receptor signaling pathway, Cytokine-cytokine receptor interaction, RIG-I-like receptor signaling pathway	IRF2, STAT, NFKAPPAB
*Reversible*	334	Cytokine-cytokine receptor interaction, Hematopoietic cell lineage, Chemokine signaling pathway	STAT5B, NFKAPPAB65, NFKB
*Augmentable*	493	RIG-I-like receptor signaling pathway, Cytosolic DNA-sensing pathway, Toll-like receptor signaling pathway	IRF2, IRF1, ISRE
*Augmentable C3-dependent*	445	RIG-I-like receptor signaling pathway, Cytosolic DNA-sensing pathway, Toll-like receptor signaling pathway	IRF2, IRF1, NFKAPPAB

^A^ According to DAVID Bioinformatics Resources 6.7 (http://david.abcc.ncifcrf.gov:8080/)

^B^ n, number of genes

^C^ C3- and CD14-dependent genes (sensitive to combined inhibition of C3 and CD14)

^D^ CD14-dependent genes (sensitive to inhibition of CD14 with anti-CD14)

^E^ C3-dependent genes (sensitive to inhibition of C3 with compstatin); see S7 Table for gene annotation enrichment analysis of specific subsets of C3- and/or C5a receptor-dependent genes

Augmented C3-DGs (n = 493) were associated with PRR signaling, cytosolic DNA-sensing and transcriptional regulation by IRFs (Table 3), which was similar to reversible CD14-DGs. Consequently, the response of 289 of these genes was reversed instead upon combined inhibition (Table 3). For comparison of different subsets of C3-DGs and C5aR-DGs, see S9 Table.

### Biological functions involved in the crosstalk between CD14 and C3

Next, we aimed to identify groups of similarly regulated IAE genes and their biological functions using cluster and gene annotation analyses (Fig. 3C and S10 Table). The resulting heat-map indicated six distinct IAE gene clusters (Fig. 3C), which we analyzed with respect to *E*. *coli* responsiveness, types of crosstalk and associated functional annotations (S10 Table).

Synergistically regulated IAE-I genes were mainly contained by clusters 1, 4 and 5. Down-regulated cluster 1 genes were associated with transcriptional regulation by peroxisome proliferator-activated receptor gamma (PPARG), while synergistically regulated IAE-I genes in general (S3 Table) as well as up-regulated cluster 5 genes (S10 Table) were associated with transcriptional regulation by the PPARG cross-regulator CCAAT/enhancer binding protein beta (CEBPB). IAEs with CD14-counteracted C3-suppressed responses (counteraction I) were mainly found in clusters 1, 3, 4 and 5. Thereby, clusters 3 and 4 contained highly up-regulated genes, which were associated with cytokine biosynthetic processes (cluster 3) or cytokine response (cluster 4) (S10 Table).

### *ERG*s in pattern recognition signaling

*ERG*s with expression fold changes above two-fold in response to *E*. *coli* and which belong to the IPA canonical pathway *Role of Pattern Recognition Receptors in Recognition of Bacteria and Viruses* are listed in Table 4. Interestingly, the majority of these genes, including cytokines, extra- and intracellular PRRs and transcription factors, were up-regulated, except for plasma membrane PRRs and the NLRC4 inflammasome, which were down-regulated. Nearly all *ERG*s contained by this pathway were reversible by combined inhibition and the majority was found to underlie an additive regulatory role of C3 and CD14 (Table 4). Only the NLRP3 inflammasome was neither sensitive to combined inhibition nor to single inhibition of C3. As another exception, complement factor B (CFB) showed a CD14-counteracted C3-suppressed response (counteraction I type of crosstalk). In fact, most of the genes were either C3-suppressed, like *C3* and *C5AR*, or C3-independent, like *C5L2* and *NFKB*. A detailed illustration of the pathway is shown in S4 Fig.

**Table 4:** Expression parameters of *ERG*s of the IPA canonical pathway *Role of Pattern Recognition Receptors in Recognition of Bacteria and Viruses*.

***ERG*s**	**Transcript ID**	***E*. *coli*** ^A^	**Combined inh.**	**CD14 inh.**	**C3 inh.**	**Type of Crosstalk (cluster)**
*Cytokines*						
**IL-6**	8131803	**129.94**	-10.69	-2.79	1.24	IAE-II (3)
**IL-12B**	8115570	**28.16**	-6.66	-4.02	2.14	IAE-II (3)
**TNF**	8118142	**9.96**	-2.53	-1.35	*n*.*s*.^B^	IAE-II (4)
**IL-1B**	8054722	**2.64**	-1.31	-1.17	*n*.*s*.	Additive
**IL-10**	7923907	**2.20**	-2.13	-1.69	*n*.*s*.	Additive
**IFNB1**	8160360	**2.18**	-1.85	-1.59	2.08	IAE-II (3)
*Pattern recognition—extracellular*						
**PTX3**	8083594	**6.63**	-3.19	-1.32	1.37	IAE-II (4)
**CFB**	8179351	**4.37**	-3.86	-4.11	2.27	Counteraction I (3)
**C3**	8033257	**2.50**	-1.29	-1.66	1.58	Additive
*Pattern recognition—plasma membrane*						
**C5L2 (GPR77)**	8029914	**-2.06**	1.29	*n*.*s*.	*n*.*s*.	Additive
**TLR1**	8099834	**-2.18**	1.97	*n*.*s*.	1.31	Additive
**CLEC7A (Dectin-1)**	7961120	**-2.26**	2.55	1.84	1.82	Additive
**C5AR1**	8029907	**-2.33**	1.30	2.04	-1.66	Additive
**TLR6**	8099841	**-2.35**	2.20	1.32	*n*.*s*.	Additive
**CD14**	8114612	**-4.67**	4.38	3.07	1.82	Additive
*Pattern recognition—intracellular*						
**IFIH1 (MDA-5)**	8056285	**4.68**	-2.27	-2.49	1.56	Additive
**OAS1**	7958884	**4.59**	-3.42	-3.49	1.33	Additive
**DDX58 (RIG-1)**	8160559	**4.03**	-2.65	-3.39	1.88	Additive
**OAS3**	7958895	**3.53**	-2.97	-2.82	1.57	Additive
**OAS2**	7958913	**3.31**	-2.72	-2.70	1.26	Additive
*Inflammasome*						
**NLRP3 (NALP3)**	7911178	**3.42**	*n*.*s*.	*n*.*s*.	*n*.*s*.	*None*
P2RX7	7959251	1.78^C^	-1.65	*n*.*s*.	*n*.*s*.	IAE-II (5)
**NLRC4 (IPAF)**	8051396	**-3.26**	3.79	1.83	2.04	Additive
*Intracellular signal transduction*						
**RIPK2 (RIP2)**	8147206	**3.05**	-1.35	-1.33	1.37	Additive
**EIF2AK2 (PKR)**	8051501	**2.60**	-2.06	-2.36	1.57	Additive
*Transcriptional regulators*						
**NFKB1 (p105)**	8096635	**2.71**	-1.77	-1.37	*n*.*s*.	IAE-II (5)
**IRF7**	7945462	**2.15**	-1.98	-1.89	1.18	Additive
**NFKB2 (p49/p100)**	7930074	**2.10**	-1.25	-1.26	*n*.*s*.	Additive

^A^ Genes with fold change (FC) expression in response to *E*. *coli* above two-fold (FC > 2; bold) listed, only, except for P2RX7; see S4 Fig. for detailed illustration of the pathway

^B^ *n*.*s*., not significant with FDR *q*-value > 0.05 (Limma) or *p*>0.05 (ANOVA; IAE cluster)

^C^ P2RX7: FC > 2 in C5-deficient patient; see S11 Table for data from a C5-deficient patient.

## Discussion

Here, we report that combined inhibition of CD14 and C3 most efficiently reduced the transcriptional response to *E*. *coli*–contained molecular patterns in a human whole blood model, which aims to mimic systemic inflammation. Combined inhibition highly significantly reduced the responses of 70% of all responding genes by on average 80% compared to the uninhibited control. Further, combined inhibition was more efficient than single inhibition of either CD14 or C3 and a prerequisite for inhibiting the response of 19% of all *E*. *coli*-responsive genes (*ERG*s).

Importantly, inflammatory responses can vary substantially among individuals. For example, cytokine production and expression of genes like tissue factor (F3) and IFIT1, differ significantly between low and high responders to LPS [18]. The microarray data of the present study were derived from two control individuals, only. In order to prove the validity of these data, supporting qPCR analyses were performed for selected genes (including F3) using samples from (i) five independent healthy individuals and (ii) the two control individuals included in the microarray. The transcriptional response to *E*. *coli* was found to underlie reasonable inter-individual variations when tested by qPCR. However, we could not distinguish between high and low responders among the individuals included in this study. Notably, all healthy individuals were chosen randomly and did not match with respect to age, gender or MBL levels [16].

C3/CD14-DGs were uniquely associated with glucose metabolism, Fcγ receptor signaling and transcriptional regulation by AP1. AP1 acts downstream of ERK, which is a central signal transducer involved in crosstalk regulation of TLR and complement signaling [3,15]. Accordingly, we identified mostly synergistic mediating roles for CD14 and C3 as well as CD14-counteracted C3-suppressive roles in the regulation of at least 11% of all *ERG*s. Suppressive effects of complement on TLR signaling have been described previously, for example for C5a on TLR induced bacterial killing in human macrophages [19], and for TLR signaling via Gαi and ERK [20]. However, we identified a substantial suppressive function of C3 reflected by increased transcriptional responses of 21% of all *ERG*s upon C3 inhibition.

Importantly, the suppressive function of C3 could be counteracted by a mediating function of CD14 (counteraction I) for at least 15% of the crosstalk-regulated genes. These genes were mostly associated with NF-κB and STAT1 regulated inflammatory responses, which are key events in the innate immunity and cytokine mediated host defense. One of the identified CD14-mediated C3-suppressed *ERG*s was the inflammasome adaptor protein PYCARD (ASC). Inflammasomes are pattern recognition complexes that mediate the maturation of IL-1 cytokine family members. Interestingly, PYCARD has also been shown to exert inflammasome-independent functions on pathogen-induced ERK activity and chemokine expression in macrophages [21]. Here, we show for the first time, that down-regulation of *PYCARD* gene expression in response to *E*. *coli* may be suppressed by complement. In addition, we found that macrophage function and inflammasome signaling were frequently involved in the complement- and CD14-mediated host response to *E*. *coli*.

In our studies we employed an inflammatory model based on human whole blood containing the thrombin-specific anticoagulant lepirudin [6]. Importantly, systemic inflammation coincides with coagulation disorders like disseminated intravascular coagulation. Also, crosstalk regulations between complement, coagulation and fibrinolysis have been observed in multiple inflammatory conditions [22]. Since only thrombin is inhibited in our model, we could detect *E*. *coli*-induced transcriptional regulation of coagulation factors, *e*.*g*., the up-regulation of plasminogen activator inhibitor-2 (*PAI-2*, *SERPINB2*) and down-regulation of thrombomodulin (*THBD*).

SerpinB2 emerges as a tightly controlled modulator of innate immunity. It has been associated with macrophage survival by preventing TLR4-induced apoptosis [23], and may mediate TLR-induced degradation of the NLRP3 inflammasome [24]. Here, we found that the up-regulation of *SerpinB2* underlay synergistic mediating roles for CD14 and C3. THBD is a multifunctional immunomodulator and a putative sepsis marker which has recently been suggested to be a component of the LPS-receptor complex CD14/TLR4/MD-2 [25]. Notably, the down-regulation of *THBD* gene expression could not be affected by combined inhibition, likely due to the opposing roles of CD14 and C3.

Among the *ERG*s, we found several suggested prognostic and diagnostic sepsis markers (reviewed in [26]), including triggering receptor expressed on myeloid cells 1 (TREM1) [27] and microRNA miR233 [28]. *TREM1* expression has been found to be decreased in sepsis but not in SIRS patients [27], indicating its potential as diagnostic marker. In our model, *TREM1* expression was CD14-dependently down-regulated in response to *E*. *coli*. MicroRNA miR223, which is a negative regulator of NF-κB and the NLRP3 inflammasome [29], was expressed at significantly higher levels in sepsis patients than in healthy controls [28]. In response to *E*. *coli* in human blood, we found that the expression of *miR223* was complement- and CD14-dependently down-regulated. This inconsistency might be explained by (i) the homogeneity of our model in contrast to polymicrobial sepsis or (ii) different inflammatory states. Our model and, thus, our observations are usually limited to the use of a single pathogen in order to study specific effects and to a single time point in order to minimize non-physiological conditions and biocompatibility issues [6]. In order to study kinetics or polymicrobial systemic inflammation, and the effect of combined inhibition on either one, other models need to be employed.

Bioenergetic switches between glucose (pro-inflammatory phase) and fatty acid (adaption phase) metabolism have been proposed to coordinate acute systemic inflammation at the epigenetic level [30]. Intriguingly, C3/CD14-DGs were associated with glucose metabolism, while for example synergistically regulated *ERG*s and augmentable C5aR-DGs were associated with fatty acid metabolism and/or the adipogenic transcription factors PPARG or CEBPB. Both factors exert their inflammatory roles in macrophages e.g., via TLR and NF-κB signaling [31,32]. Anti-inflammatory PPARG has been reported to mediate proteasomal degradation of NF-κB [31], while pro-inflammatory, IL-6-inducible CEBPB regulates Fcγ receptor-mediated inflammatory responses, which can be further enhanced by C5a [32]. Both *PPARG* and *CEBPB* themselves were down-regulated in response to *E*. *coli* and underlay crosstalk with either C5-dependent dominant suppressive effects of C3 (counteraction II) or C5-independent synergistic roles for C3 and CD14, respectively. To our knowledge, this is the first report suggesting that CD14 and complement crosstalk may coordinate bioenergetic processes in acute inflammation, and that CEBPB and PPARG may be involved in this scenario.

## Materials and Methods

### *Ex vivo* model of Gram-negative bacteria-induced inflammation in human whole blood

The *ex vivo* model of inflammation [6] as well as detailed experimental procedures, blood sample generation and description of the donors have been previously described [16]. Briefly, whole blood samples from healthy donors (n = 2) were incubated for 120min at 37°C in NUNC cryo tube vials (Roskilde, Denmark) in presence of 1x10^6^/mL or 5x10^6^/mL heat-inactivated *E*. *coli* (strain LE392; ATCC 33572, Manassas, VA) and inhibitors. The setup gave rise to four independent observations for each of the five experimental conditions (see Statistical analyses—Limma). Complement activation at the level of C3 was blocked by compstatin (Ac-I[CV(1MeW) QDWGAHRC]T) (kindly provided by Prof. John Lambris) [33]. CD14 was inhibited by a blocking mouse anti-human CD14 F(ab’)_2_ antibody fragment (clone 18D11) or a F(ab’)_2_ control (clone BH1) (Diatec AS, Oslo, Norway). Blood was prevented from coagulation by the highly specific thrombin inhibitor lepirudin (50 μg/mL; Refludan, Pharmion, Copenhagen, Denmark).

### DNA microarray analysis

RNA was isolated from whole blood (see S1 Materials and Methods). Then, 150ng of total RNA was subjected to cDNA synthesis using the GeneChip Whole Transcript (WT) Sense Target Labelling Assay (Affymetrix; Manual: P/N701880 Rev.4) and hybridized with GeneChip Gene 1.0 ST Array (Affymetrix). Staining and washing was performed using the GeneChip Hybridization, Wash and Stain Kit (Affymetrix; P/N 900720) on the Fluidics Station 450 using protocol FS450_0007. Signal values (SV) from all chips were log-transformed (log_2_), normalized by background reduction using Robust Multichip Analysis (RMA; Partek Genomics Suite software), and filtered using a threshold of log_2_SV = 4. Transcripts which passed the procedure (n = 19,695) were subjected to downstream statistical analyses. Pearson’s correlation analysis (RMA) was performed (S5 Fig.) using filtered data in order to check for possible outliers.

The microarray expression data discussed in this paper have been deposited in NCBI’s Gene Expression Omnibus (GEO) [34] and are accessible with GEO Series accession number GSE55537 (http://www.ncbi.nlm.nih.gov/geo/query/acc.cgi?acc=GSE55537).

### Quantitative polymerase chain reaction, qPCR

The microarray data were verified by qPCR for seven *ERG*s (CD14, CXCL10, IL-1A, IL-6, miR155, SerpinB2, tissue factor (F3)) and TLR4 using either the same RNA material (technical verification; see S1 Materials and Methods and S6 Fig.) or RNA isolated from blood of five independent healthy individuals (biological verification; see below). Freshly drawn venous blood from five healthy donors was incubated, in accordance with the whole blood model, for 120min at 37°C in presence or absence of 1x10^6^/mL heat-inactivated *E*. *coli* and/or inhibitors of C3 and CD14. Total RNA was isolated using the ABI PRISM 6100 Nucleic Acid PrepStation and the Applied Biosystems AB6100 total RNA chemistry (Life Technologies, Applied Biosystems, Foster City, CA, USA). cDNA synthesis was performed using a High-Capacity cDNA Reverse Transcription Kit from Applied Biosystems and 0.9ng/μl total RNA. qPCR was performed in triplicates as a gene-maximization approach in MicroAmp Fast 96-well plates using predesigned gene-specific primer, FAM-labeled minor groove-binding probes and TaqMan Fast Universal PCR master mix, all provided by Applied Biosystems (Life Technologies). Data were analysed on the 7500 Fast Real-Time PCR System using the 7500 Software from Applied Biosystems. Relative quantification of gene expression was performed with the comparative method of Livak and Schmittgen (RQ = 2^(-ΔΔCt)^) [35]. As reference genes beta-2-microglobulin (B2M; gene expression assay ID: Hs99999907_m1), large ribosomal protein P0, (RPLP0; Hs99999902_m1), and TATA box-binding protein (TBP; Hs00427620_m1) were chosen. The arithmetic mean of their expression data was used for relative quantification of the selected target genes: CD14 (Hs02621496_s1), CXCL10 (Hs01124251_g1), IL-1A (Hs00174092_m1), IL-6 (Hs00985639_m1), miR155HG (Hs01374569_m1), SerpinB2 (Hs01010736_m1), F3 (Hs01076029_m1) and TLR4 (Hs00152939_m1). As an indicator for inter-run variation, B2M expression was assayed using the same sample on each plate. Its quantification cycle at threshold (Ct) differed only slightly between plates (21.76±0.23), indicating negligible technical variation.

### Statistical analyses—Limma

Linear models for microarray data (Limma; Bioconductor) [36,37] was used to calculate differential gene expression from the filtered microarray data set. A false discovery rate (FDR) (multiple testing adjusted *p*-values (*q*-values)) [38] of 5% was used as significance threshold. Differential expression was determined for: uninhibited (presence of *E*. *coli*) versus spontaneous activation (absence of *E*. *coli*), and inhibited (presence of *E*. *coli* and either compstatin, anti-CD14 or a combination of both) versus uninhibited activation. Subsequently, the log_2_-transformed fold change (log_2_FC) expression estimates from four replicates, represented by two days and two healthy donors were combined as follows: data of day one and day two for each donor were pooled before the mean of both pools was calculated. Negative and positive fold change (FC) values for uninhibited *E*. *coli* response were interpreted as transcriptional down- and up-regulation. Negative and positive FC values for the inhibited response for up-regulated *ERG*s were interpreted as reversion and augmentation, respectively; and the other way around for down-regulated *ERG*s. Data from the microarray analyses for the initial state (0 min incubation in absence of *E*. *coli*) and the inhibitor controls were included in Pearson’s correlation analysis (see DNA Microarray analysis) and for comparison with qPCR data (S6 Fig.), only.

Of the 19,695 transcripts included in the analysis, 2335 had an FDR *q*-value below 5% for the uninhibited *E*. *coli* response, and were designated *E*. *coli*-responsive genes (*ERG*s). For all subsequent analysis, only the datasets for *ERG*s were tested for statistical significance.

### Statistical analyses—ANOVA

Analysis of variance (ANOVA) was used to define the subset of *ERG*s putatively regulated by crosstalk between complement and CD14. In the ANOVA model, we included individual effects of complement and CD14, as well as an interaction effect between the two. Crosstalk was declared for an *ERG* when the ANOVA analysis resulted in a significant interaction effect (IAE), *i*.*e*. when the sum of effects of single inhibitions with compstatin or anti-CD14 was significantly different from the effect of combined inhibition with compstatin and anti-CD14 (*p*<0.05). The statistical tests were based on normalized and filtered raw data for the 2335 *ERG*s in control conditions. In total, 1505 *ERG*s showed a significant anti-CD14 effect, 712 showed a compstatin effect and 499 genes showed both. A significant interaction effect was observed for 251 *ERG*s.

### Cluster analysis

Hierarchical cluster analyses were performed using the heatmap.2 function in the gplots package [39] in R with default method parameters (Euclidian distance and complete linkage) based on Limma-derived differential expression data (log_2_FC) for comparison of *E*. *coli* responses in the absence versus presence of inhibitors of genes with significant ANOVA-based interaction effects (n = 251) (Fig. 3C).

### Functional annotation analyses

Data were analyzed through the use of QIAGEN’s Ingenuity “Pathway Analysis” (IPA, QIAGEN Redwood City, www.qiagen.com/ingenuity) and the Database for Annotation, Visualization and Integrated Discovery (DAVID) [40]. Gene ontology annotations associated with top regulated genes (S5 Table, S6 Table, S7 Table and S8 Table) were retrieved from UniProtKB-GOA [41].

### Experiments using C5-deficient blood

All experiments were also performed using a CD88-specific C5a receptor (C5aR) antagonist (C5aRA) AvF[OPdChaWR] (kindly provided by Prof. John Lambris) [42] or blood from a C5-deficient donor without (C5D) or with (C5DR) reconstitution with 80μg/mL purified recombinant human C5 (Quidel, San Diego, CA). In order to increase statistical power, samples from day one (1x10^6^/mL *E*. *coli*) for C5D and C5DR were analysed in technical duplicates by DNA microarray technology. See S1 Materials and Methods for a description of the analyses, and S1 Fig., S3 Fig., S1 Table, S7 Table, S8 Table and S11 Table for the results.

### Ethics Statement

The study has been approved by the Regional Ethical Committee (REC) of the Northern Norway Regional Health Authority. Written informed consent was obtained from all donors.

## Supporting Information

S1 Fig *E*. *coli* response in a C5-deficient background and inhibition of CD14 and C3. (TIF)

S2 Fig Differential C3- and C5a receptor-dependencies. (TIF)

S3 Fig Crosstalk between CD14 and C3 in a C5-deficient background (C5D). (TIF)

S4 Fig *ERG*s in the IPA canonical pathway *Role of Pattern Recognition Receptors in Recognition of Bacteria and Viruses*. (TIF)

S5 Fig Pearson’s Correlation analyses of microarray data. (TIF)

S6 Fig Verification of microarray data by qPCR. (TIF)

S1 File IAE-I gene list. (XLSX)

S2 File IAE-II gene list. (XLSX)

S3 File Additive effect gene list. (XLSX)

S1 Materials and Methods (DOCX)

S1 Table *ERG*s and their sensitivity to inhibition of complement and CD14 in a C5-deficient background. (DOCX)

S2 Table *ERG*s and their sensitivity to single inhibition of C5a receptor 1 (CD88). (DOCX)

S3 Table Types of crosstalk between CD14 and C3 signaling in response to *E*. *coli* (ANOVA, *p*<0.05). (DOCX)

S4 Table Gene annotation enrichment analysis of *ERG*s in human whole blood using DAVID (*p*<0.05). (DOCX)

S5 Table Top ten up-regulated *ERG*s (FC, FDR *q*-value < 0.05). (DOCX)

S6 Table Top ten down-regulated *ERG*s (FC, FDR *q*-value < 0.05). (DOCX)

S7 Table Top ten up-regulated *ERG*s and their responses upon C5-deficiency (FC, FDR *q*-value < 0.05). (DOCX)

S8 Table Top ten down-regulated *ERG*s and their responses upon C5-deficiency (FC, FDR *q*-value < 0.05). (DOCX)

S9 Table Gene annotation enrichment analysis of specific subsets of C3- and/or C5aR-dependent *ERGs* (DAVID; *p*<0.05). (DOCX)

S10 Table Clusters of IAE genes, associated types of crosstalk and functional annotations (DAVID, *p*<0.1). (DOCX)

S11 Table *ERG*s of the IPA canonical pathway *Role of Pattern Recognition Receptors in Recognition of Bacteria and Viruses*—Expression data from a C5-deficient background. (DOCX)
