# Supplementary figures and images for: CD14 and Complement Crosstalk and Largely Mediate the Transcriptional Response to Escherichia coli in Human Whole Blood as Revealed by DNA Microarray
Source: PLoS One. 2015 Feb 23;10(2):e0117261. doi: 10.1371/journal.pone.0117261 (PMC4338229; doi:10.1371/journal.pone.0117261)

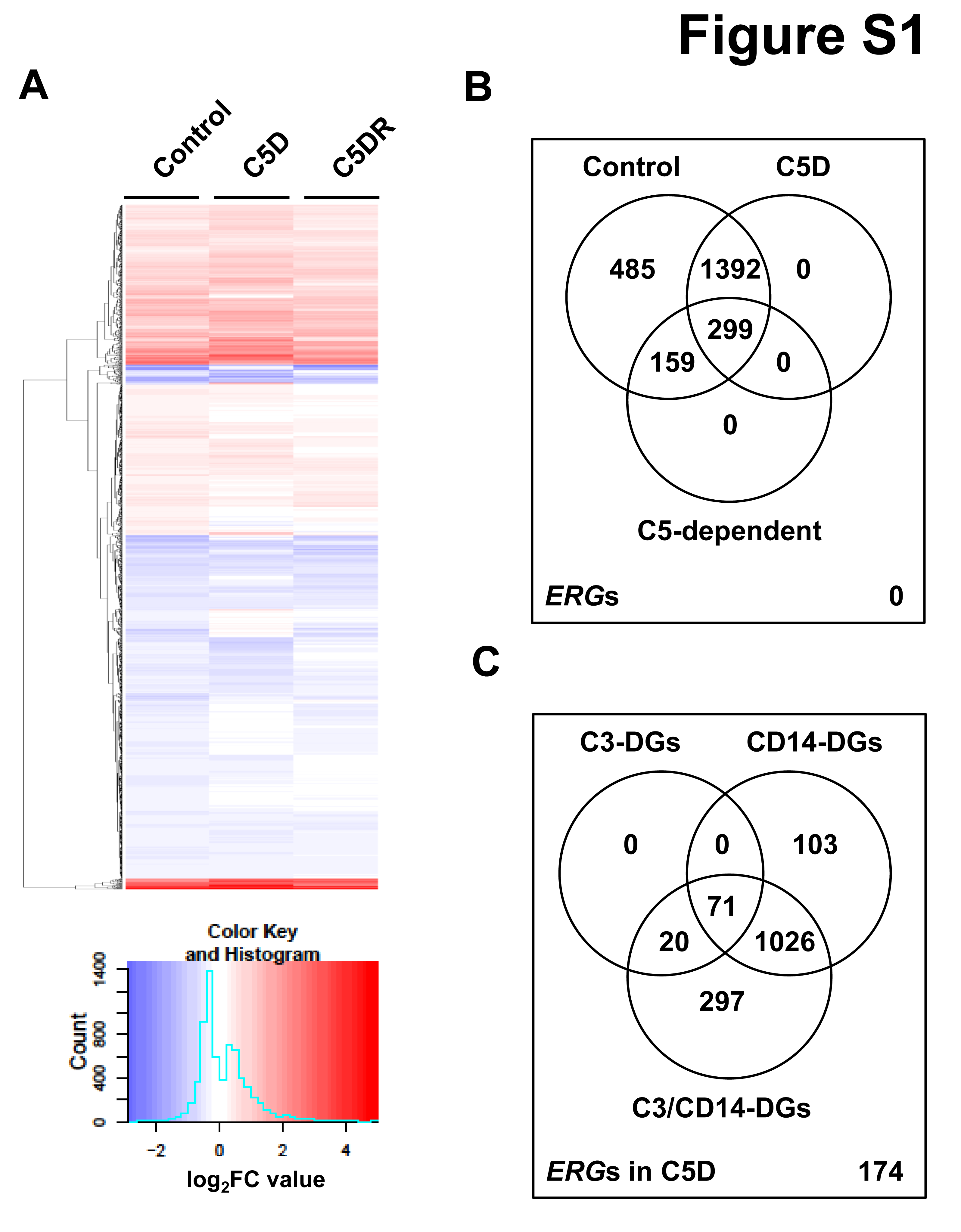

Supplement: S1 Fig — (TIF) [file pone.0117261.s001.tif]

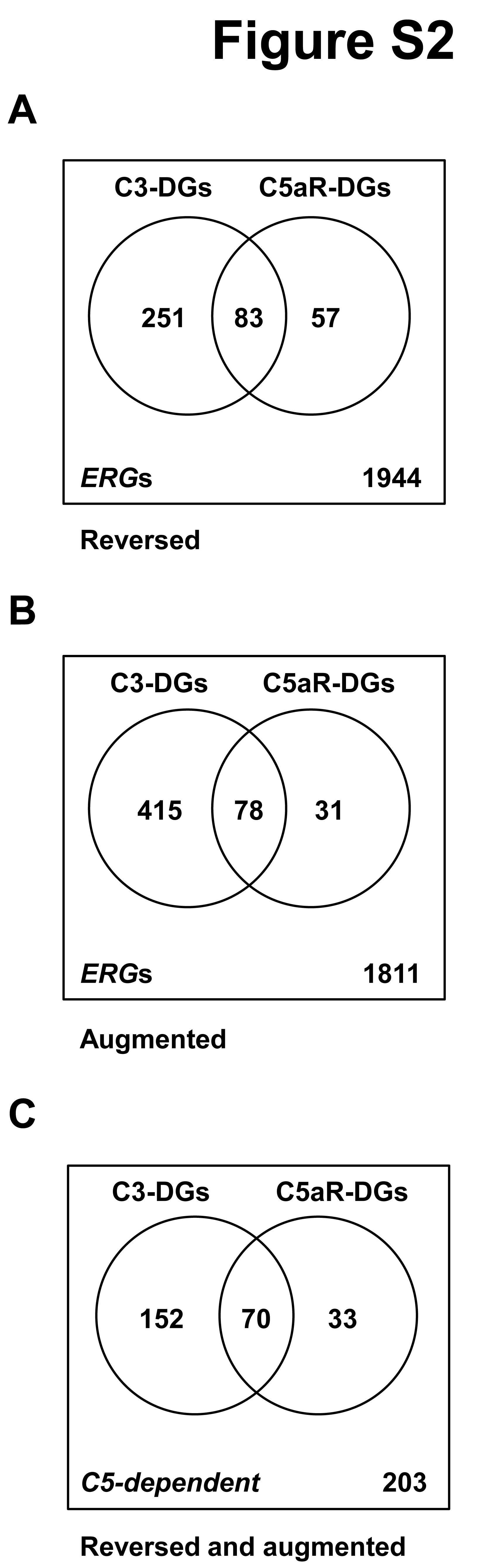

Supplement: S2 Fig — (TIF) [file pone.0117261.s002.tif]

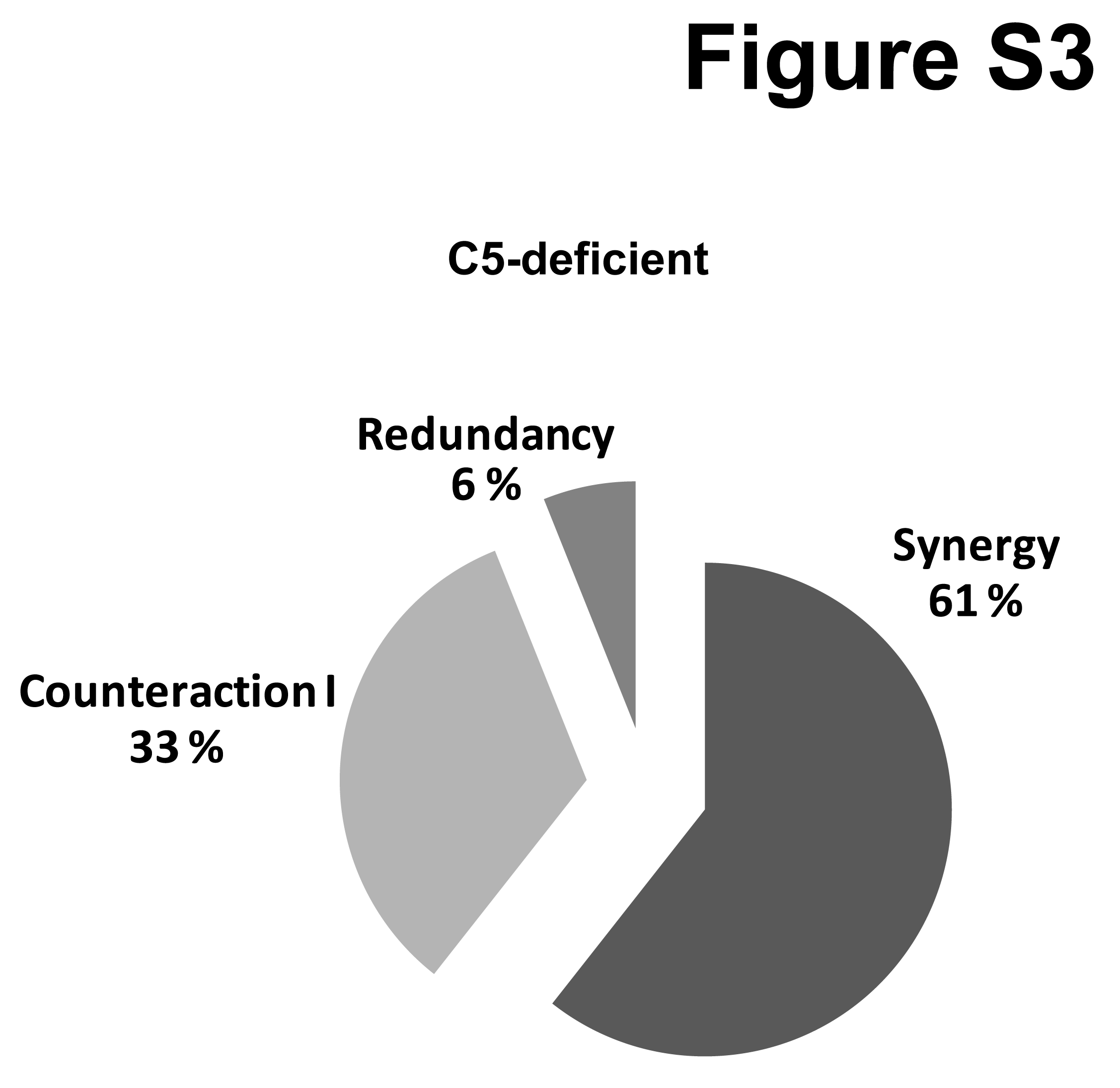

Supplement: S3 Fig — (TIF) [file pone.0117261.s003.tif]

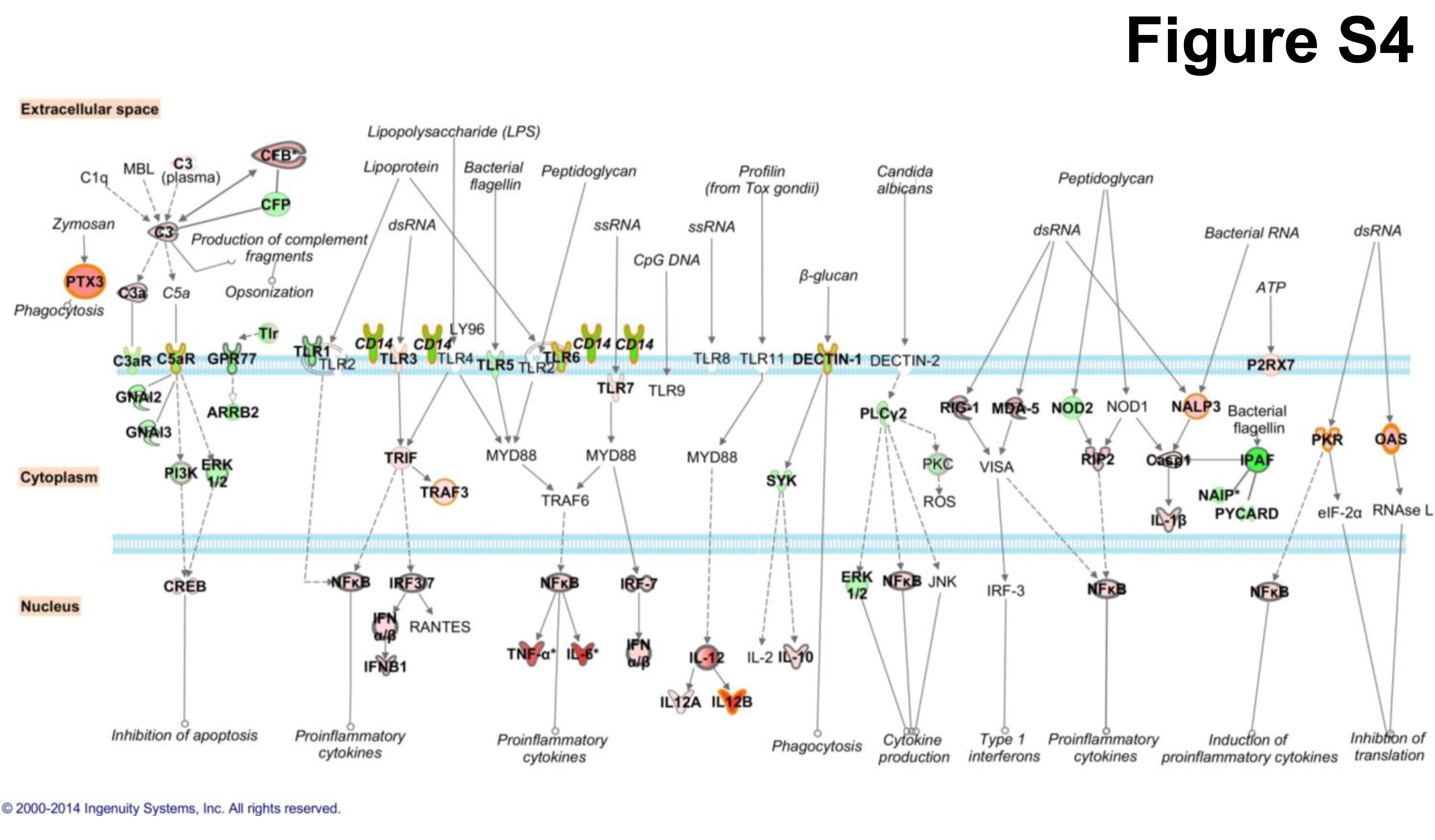

Supplement: S4 Fig — (TIF) [file pone.0117261.s004.tif]

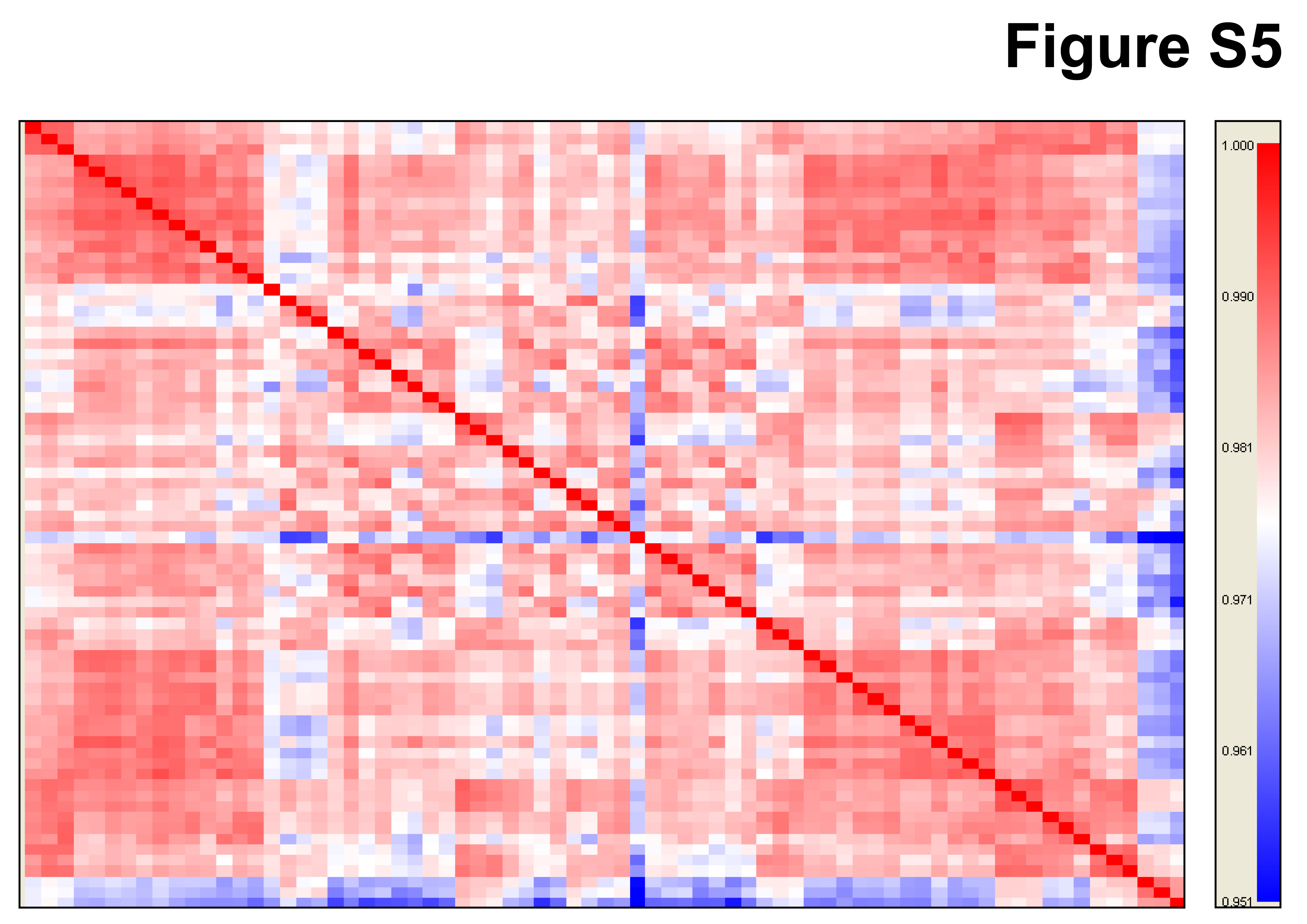

Supplement: S5 Fig — (TIF) [file pone.0117261.s005.tif]

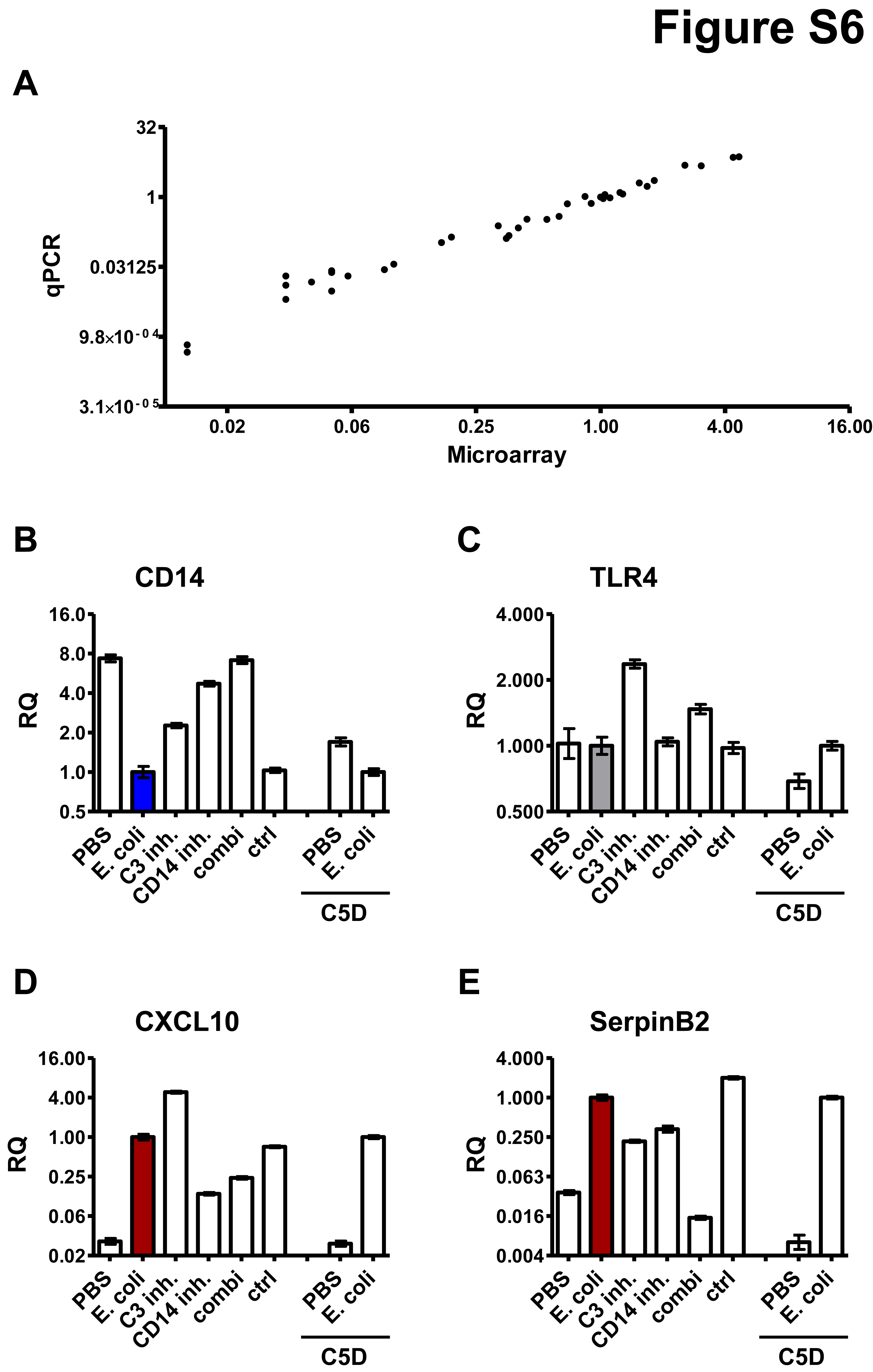

Supplement: S6 Fig — (TIF) [file pone.0117261.s006.tif]
