# Supplementary material for: CD14 and Complement Crosstalk and Largely Mediate the Transcriptional Response to Escherichia coli in Human Whole Blood as Revealed by DNA Microarray
Source: PLoS One. 2015 Feb 23;10(2):e0117261. doi: 10.1371/journal.pone.0117261 (PMC4338229; doi:10.1371/journal.pone.0117261)
Supplement: S1 Materials and Methods — (DOCX) [file pone.0117261.s010.docx]

**Supplementary Materials and Methods**

*Sample preparation for Microarray analysis*

Complement- and CD14 inhibitors were preincubated with lepirudin-blood for 7 min at 37^o^C at final concentrations of 25 mM compstatin, 10 mM C5aR antagonist and 50 µg/mL anti-CD14. Subsequently, 1 x 10^6^/mL (day 1) or 5 x 10^6^/mL (day 2) heat inactivated *Escherichia coli* (strain LE392; ATCC 33572, Manassas, VA) or phosphate buffered saline (PBS) with calcium and magnesium (Sigma, St. Louis, MO) were added and incubation was proceeded for 120 min at 37^o^C. For each sample, total RNA was isolated from two ml human blood, lysed in three mL 1x nucleic acid purification lysis buffer (Life Technologies, Applied Biosystems, Foster City, CA, USA; PN4305895). The purification was performed batch-wise with 16 to 18 samples per plate following the standard procedure for ABI PRISM 6100 Nucleic Acid PrepStation using Applied Biosystems AB6100 total RNA chemistry. Total RNA was recovered in 150 µL nucleic acid purification elution solution (Applied Biosystems; PN4305893) per well and subsequently precipitated by addition of 2.5 volumes ethanol (96 %) and 10 % 3 M sodium acetate. After incubation at -70^o^C overnight, the RNA was washed in 70 % ethanol, dried and recovered in 60 µL elution solution. The RNA was of high quality with an average RNA integrity number of 8.8 when analyzed on a 2100 Bioanalyzer (Agilent). RNA concentration was determined using a Nanodrop system. The average RNA yield was 2.2 µg per mL blood. Of each sample, 150 ng total RNA in concentrations of minimum 50 ng/µL was subjected to cDNA synthesis and amplification followed by *in vitro* transcription, clean-up and labeling using Affymetrix^®^ GeneChip^®^ Whole Transcript (WT) Sense Target Labelling Assay (Manual: P/N701880 Rev.4) prior to *DNA Microarray analyses*.

*Technical verification of DNA microarray data by qPCR*

DNA microarray data of the healthy controls (two healthy donors and two days; n=4) and a C5-deficient patient (C5D) (two days; n=2) were verified by qPCR for seven selected target genes (IL-6, CD14, CXCL10, SerpinB2, miR155, IL-1A, TLR4) using three reference genes (B2M, RPLP0, TBP) and the same RNA material. Relative expression was determined for the following experimental conditions: absence of *E. coli* (PBS), presence of *E. coli* only (*E. coli*) and presence of *E. coli* and inhibitor of C3, CD14, both inhibitors (combi) or inhibitor controls (ctrl). According to 2100 Bioanalyzer (Agilent), RNA samples had an RNA integrity number (RIN) of around 7 at the time of reverse transcription (RT) and showed no considerable degradation. RT and qPCR were performed as described in the Material and Methods section in the main article. As reference genes, beta-2-microglobulin (B2M; gene expression assay ID: Hs99999907_m1), ribosomal protein, large, P0, (RPLP0; Hs99999902_m1), and TATA box-binding protein (TBP; Hs00427620_m1) were chosen. The arithmetic mean of their expression data was used for relative quantification of the selected target genes: IL-6 (Hs00985639_m1), CD14 (Hs00169122_g1), CXCL10 (Hs00171042_m1), SerpinB2 (Hs01010736_m1), MIR155HG (Hs01374569_m1), IL-1A (Hs00174092_m1) and TLR4 (Hs00152939_m1). As an indicator for inter-run variation, B2M expression was assayed using the same sample on each plate. Its quantification cycle at threshold (Ct) differed only slightly between plates (22.27 ± 0.31), indicating negligible technical variation.

*Statistical analyses for data from C5-deficient patient - Limma*

Differential expression was determined between the following experimental conditions for the C5-deficient background without (C5D) or with C5 reconstitution (C5DR): uninhibited (presence of *E. coli*) versus spontaneous activation (absence of *E. coli*) and inhibited (presence of *E. coli* and either C3 inhibitor, CD14 inhibitor, a combination of both, or C5aR inhibition) versus uninhibited. Also, data were compared between healthy donor and C5D, healthy donor and C5DR, and C5D and C5DR. Prior to these analyses, the raw array data from three replicates (datasets) for C5D or C5DR represented by two days and one technical replicate of day 1 were combined as follows: a fold change estimate was calculated from the pooled data of the replicates of day 1 and from the data of day 2, before the mean of both estimates was calculated. The technical replicate, which substituted for the lack of biological ones, contributed to higher correlation between the datasets of C5D, and thus to a higher statistical significance compared to the healthy donors. For uninhibited expression versus spontaneous expression in absence of *E. coli* in C5D, 3237 of the 19695 transcripts included in the analysis had an FDR *q*-value below 5 %. Similarly, 1691 of the 2335 *ERG*s from healthy donors had an FDR *q*-value below 5 % also for the *E. coli* response in C5D (see Supplementary figure S1 Figure, panel B).

*Cluster analysis – Effect of C5-deficiency*

Hierarchical cluster analyses were performed using the heatmap.2 function in the gplots package (30) in R with default method parameters (Euclidian distance and complete linkage) based on Limma-derived differential expression data (log_2_FC) for comparison of *E. coli* responses (n = 2335) in healthy donors with those in blood from the C5-deficient patient in absence (C5D) and presence of C5 reconstitution (C5DR) (Supplementary figure S1 Figure, panel A).

*Pathway analyses using Ingenuity Pathway Analysis (IPA)*

Affymetrix transcript cluster ID, fold change values and FDR *q*-values for the 2335 *E. coli*-responsive genes (*ERG*) were submitted to Ingenuity pathway analysis (application build 131335, January 2012; content version 11904312, December 2011). Including data for all contrasts, except for initial state (0 min incubation in the absence of *E. coli*) versus spontaneous activation (120 min incubation in absence of *E. coli*) for both control and C5D, 2156 *ERG*s could be mapped to the Ingenuity knowledge base. For core analysis, the filter was set for direct and indirect interactions, human-specific, experimentally observed data for immune cells, only, concerning both, molecules and relationships (stringent filter). For network analysis, 70 molecules per network were requested. Only genes with FDR *q*-values below 5 % contributed to core analyses. The output of core analyses included top networks, top canonical pathways and top molecules.

*Gene annotation enrichment and pathway analyses using DAVID*

Gene annotation enrichment analysis using the interactive database DAVID Bioinformatics Resources 6.7 (http://david.abcc.ncifcrf.gov:8080/) [2,3] and its associated databases KEGG pathways, Gene ontology (GO) and University of California Santa Cruz, USCS, Genome Browser (http://genome.ucsc.edu/cgi-bin/hgTables) were employed to find biochemical pathways, molecular functions and transcription factors (TFBS), respectively, which were most frequently associated with certain gene subsets. The gene subsets were submitted to DAVID as lists of Affymetrix transcripts cluster IDs. By default, the search of GO returned associations with molecular function (GO_MF_fat) that were filtered for the broadest terms for each category. The results were considered statistically significant when *p* < 0.05, if not indicated differently.

*Data presentation*

Venn diagrams were based on lists of Affymetrix transcript IDs, which were compared using the online interactive tool Venny (<http://bioinfogp.cnb.csic.es/tools/venny/index.html>). For data presentation in graphs, the GraphPad Inc. software GraphPad Prism V5.03 was used. The diagrams in Figure 2B and Supplementary figure S3 Figure were generated using Microsoft Excel 2007.

Reference List

1. Livak KJ, Schmittgen TD (2001) Analysis of relative gene expression data using real-time quantitative PCR and the 2(-Delta Delta C(T)) Method. Methods 25: 402-408. 10.1006/meth.2001.1262 [doi];S1046-2023(01)91262-9 [pii].

2. Huang dW, Sherman BT, Lempicki RA (2009) Systematic and integrative analysis of large gene lists using DAVID bioinformatics resources. Nat Protoc 4: 44-57. nprot.2008.211 [pii];10.1038/nprot.2008.211 [doi].

3. Huang dW, Sherman BT, Lempicki RA (2009) Bioinformatics enrichment tools: paths toward the comprehensive functional analysis of large gene lists. Nucleic Acids Res 37: 1-13. gkn923 [pii];10.1093/nar/gkn923 [doi].
