# Supplementary material for: CD14 and Complement Crosstalk and Largely Mediate the Transcriptional Response to Escherichia coli in Human Whole Blood as Revealed by DNA Microarray
Source: PLoS One. 2015 Feb 23;10(2):e0117261. doi: 10.1371/journal.pone.0117261 (PMC4338229; doi:10.1371/journal.pone.0117261)
Supplement: S1 Table — (DOCX) [file pone.0117261.s011.docx]

**S1 Table.** *ERG*s and their sensitivity to inhibition of complement and CD14 in a C5-deficient background.

| **Category** | **Number of transcripts**^A^ | | |
| --- | --- | --- | --- |
|  | **Total** | **Reversible** | **Augmentable** |
| ***ERG*s** | 1691^B^ | 1517^C^ | 11^D^ |
| *Up-regulated* | 896 | 819 | 10 |
| *Down-regulated* | 795 | 698 | 1 |
| *FC > 2* ^E^ | 394 | 391 | 0 |
| **C3/CD14-DG**^F^ | 1419 | 1414 | 5 |
| **CD14-DG**^G^ | 1202 | 1200 | 2 |
| **C3-DG**^H^ | 140 | 91 | 49 |
| **C5aR-DG**^I^ | 1 | 1 | 0 |

^A^ Genes may count redundantly in different categories.

^B^ Of C5D *ERG*s, 163 were neither reversible (n=1517) nor augmentable (n=11), and, therefore, defined as independent of CD14, C3 and C5a receptor (CD88).

^C^ Reversed by at least one inhibitory strategy, including inhibition with a C5a receptor antagonist

^D^ Not reversible at all

^E^ Fold change expression (FC) in response to *E. coli* above two-fold (FC > 2)

^F^ C3- and CD14-dependent genes (sensitive to combined inhibition of C3 and CD14)

^G^ CD14-dependent genes (sensitive to inhibition of CD14 with anti-CD14)

^H^ C3-dependent genes (sensitive to inhibition of C3 with compstatin)

^I^ C5aR-dependent genes (sensitive to inhibition of C5a receptor 1 (CD88) with C5aR antagonist)
