# Supplementary material for: CD14 and Complement Crosstalk and Largely Mediate the Transcriptional Response to Escherichia coli in Human Whole Blood as Revealed by DNA Microarray
Source: PLoS One. 2015 Feb 23;10(2):e0117261. doi: 10.1371/journal.pone.0117261 (PMC4338229; doi:10.1371/journal.pone.0117261)
Supplement: S2 Table — (DOCX) [file pone.0117261.s012.docx]

**S2 Table.** *ERG*s and their sensitivity to single inhibition of C5a receptor 1 (CD88).

| **Category** | **Number of transcripts** | | |
| --- | --- | --- | --- |
|  | **Total** | **Reversible** | **Augmentable** |
| **C5aR-DG^A^** | 249 | 140 | 109 |

^A^ C5aR-dependent genes (sensitive to inhibition of C5a receptor 1 (CD88))
