# Supplementary material for: CD14 and Complement Crosstalk and Largely Mediate the Transcriptional Response to Escherichia coli in Human Whole Blood as Revealed by DNA Microarray
Source: PLoS One. 2015 Feb 23;10(2):e0117261. doi: 10.1371/journal.pone.0117261 (PMC4338229; doi:10.1371/journal.pone.0117261)
Supplement: S3 Table — (DOCX) [file pone.0117261.s013.docx]

**S3 Table.** Types of crosstalk between CD14 and C3 signaling in response to *E. coli* (ANOVA, *p*<0.05).

| **Type of crosstalk** | **n**^A^ | **Gene Symbols** | **Transcription factor**^B^ |
| --- | --- | --- | --- |
| **1. Interaction effect, IAE** | 251 | *See 1.1 and 1.2* | AP1, CEBPB, NFKB |
| **1.1 IAE-I** | 110 | See S1 Supporting File: IAE-I Gene List |  |
| ***1.1.1 Synergy*** | 59 | **UP:** ABCA1, ACSL5, AEN, ANXA5, ARFGAP3, ATP13A3, ATP2B1, CCRL2^C^, CCRL2, CHST7, DKFZp686O24166, EDN1, ELOVL7^D^, FAM115C, FLT1, INSIG1, IRAK2^E^, LCP2, NR4A3, NUP188, PFKFB3, SERPINB2, SNX9, SOCS3  **DOWN:** 8100125^F^, ACOX1, AMICA1, ARRB1, ATG16L2, BTK, C10orf54, CEBPB, CLEC10A, CREB5, CTSH, EVI2B, F11R, FLJ10357, GABARAP, GPR177, HLA-DMB^C^, HLA-DMB, IFNGR1, KIF13A, KIAA0513, KIAA1949, LTA4H, MOSPD2, PGD, PLXNB2, PRCP, RAB3D, RNF41, SEPX1, SULF2, SYK, TMEM71, TNFRSF10C, USP32 | CEBPB, MAX, CEBP;  *Icosanoid metabolic process, fatty acid metabolic process, secretion by cell* |
| ***1.1.2 Counteraction I*** ^G^ | 38 | **UP:** 8017096^F^, 8017098^F^, 8083933^F^, 8172266^F^, 8172270^F^, ADPRH, C15orf48, CCL20, CCL8, CD44, CFB^C^, CFB, CFB, CH25H, DDX60L, FMNL3, FNIP2, IL1A, IRG1, MAP3K8, PPM1K, PSMA6, PTPRJ, RHOU, SLC43A3, TOR1B, UNQ6228  **DOWN:** C22orf9, CBL, CCR2^C^, CLMN, CPNE2, DNTTIP1, FAM135A, PSTPIP1, PYCARD, TUBA1B, ZFP36L2 | NFKB, STAT1, NFKAPPAB;  *Inflammatory response, response to wounding,*  *defense response* |
| *1.1.3 Counteraction II* ^H^ | 7 | **DOWN:** HBEGF, CDCP1, MITF, NAB2, PPARG, SPP1, STK38L | *n.s.* |
| *1.1.4 Counteraction III* ^I^ | 1 | **UP:** DOT1L | *n.s.* |
| *1.1.5 Redundancy* | 2 | **UP:** C9orf30; **DOWN:** TNFRSF12A | *n.s.* |
| *1.1.6 Negative redundancy* | 3 | **DOWN:** CCR2^C^, KIAA1598, SLC2A9 | *n.s.* |
| **1.2 IAE-II** | 141 | See S2 Supporting File: IAE-II Gene List | AP1, NFKAPPAB, MRF2 |
| **2. Additive effect** | 1480 | See S3 Supporting File: Additive effect Gene List | IRF2, AML1, ELK1 |

^A^ n, number of genes

^B^According to DAVID Bioinformatics Resources 6.7; For Synergy and Counteraction I, associated gene ontology (Biological Processes) is given as well. *n.s.*, no significant hit

^C^ The same gene was detected with two (CCRL2, HLA-DMB, CCR2) or three (CFB) different probe sets, of which each is associated with a non-redundant Affymetrix transcript ID.

^D^ Doubled underlined, IAE-I genes which changed their crosstalk type upon C5-deficiency.

^E^ Underlined, IAE-I genes which kept their crosstalk type upon C5-deficiency. The remaining IAE-I genes lost their interaction effect upon C5-deficiency

^F^ The Affymetrix transcript ID is given due to the lack of official gene symbol.

^G,H,I^ Counteractions are defined as: CD14 counteracts blocking function of C3 (G), C3 counteracts mediating function of CD14 (H), C3 counteracts blocking function of CD14 (I).
