# Supplementary material for: CD14 and Complement Crosstalk and Largely Mediate the Transcriptional Response to Escherichia coli in Human Whole Blood as Revealed by DNA Microarray
Source: PLoS One. 2015 Feb 23;10(2):e0117261. doi: 10.1371/journal.pone.0117261 (PMC4338229; doi:10.1371/journal.pone.0117261)
Supplement: S4 Table — (DOCX) [file pone.0117261.s014.docx]

**S4 Table.** Gene annotation enrichment analysis of *ERG*s in human whole blood using DAVID^A^ (*p*<0.05).

| **Category**  *Subcategory* | **n**^B^ | **KEGG Pathway** | **n** |  | **Molecular Function** | **n** |  | **Transcription**  **factor** | **n** |
| --- | --- | --- | --- | --- | --- | --- | --- | --- | --- |
| ***ERGs*** | 2335 | Cytokine-cytokine receptor interaction | 82 |  | Cytokine binding | 38 |  | IRF2 | 802 |
|  |  | NOD-like receptor signaling pathway | 30 |  | Cytokine activity | 54 |  | AP1 | 1138 |
|  |  | Chemokine signaling pathway | 59 |  | GTPase activator activity | 51 |  | AML1 | 1445 |
| *Below two-fold* | 1973 | Chemokine signaling pathway | 42 |  | GTPase regulator activity | 69 |  | ELK1 | 836 |
|  |  | Lysosome | 30 |  | Nucleoside-triphosphatase regulator activity | 70 |  | AP1 | 983 |
|  |  | Hematopoietic cell lineage | 22 |  | Cytokine binding | 27 |  | IRF2 | 688 |
| *Above two-fold* | 362 | Cytokine-cytokine receptor interaction | 40 |  | Cytokine activity | 34 |  | IRF1 | 77 |
|  |  | Toll-like receptor signaling pathway | 19 |  | Chemokine receptor binding | 15 |  | AP2α | 3 |
|  |  | NOD-like receptor signaling pathway | 13 |  | Chemokine activity | 14 |  | IRF2* | 115 |
| *Reversible* ^C^ | 1892 | Cytokine-cytokine receptor interaction | 78 |  | Cytokine activity | 52 |  | IRF2 | 662 |
|  |  | Chemokine signaling pathway | 54 |  | Cytokine binding | 36 |  | AP1 | 941 |
|  |  | Toll-like receptor signaling pathway | 35 |  | Enzyme activator activity | 63 |  | AML1 | 1192 |
| *Augmentable* ^D^ | 105 | RIG-I-like receptor signaling pathway* | 3 |  | Integrin binding* | 3 |  | MAX | 25 |
|  |  | Fc gamma R-mediated phagocytosis* | 3 |  | Protein deacetylase activity* | 2 |  | TATA | 56 |
|  |  |  |  |  | Histone deacetylase activity* | 2 |  | ARNT | 48 |
| *Independent* ^E^ | 338 | RIG-I-like receptor signaling pathway | 5 |  | Ligase activity, forming carbon-nitrogen bonds | 10 |  | EGR1 | 32 |
|  |  | NOD-like receptor signaling pathway | 4 |  | Enzyme binding | 15 |  | CREB | 122 |
|  |  | Endocytosis | 7 |  | Acid-amino acid ligase activity | 8 |  | AHR | 96 |

^A^ According to DAVID Bioinformatics Resources 6.7 (http://david.abcc.ncifcrf.gov:8080/)

^B^ n, number of associated *ERG*s (redundancy may occur); * *p* < 0.1

^C^ Reversed by at least one inhibitory strategy

^D^ Not reversible at all

^E^ Neither reversible or augmentable by any inhibitory strategy used in this study
