# Supplementary material for: CD14 and Complement Crosstalk and Largely Mediate the Transcriptional Response to Escherichia coli in Human Whole Blood as Revealed by DNA Microarray
Source: PLoS One. 2015 Feb 23;10(2):e0117261. doi: 10.1371/journal.pone.0117261 (PMC4338229; doi:10.1371/journal.pone.0117261)
Supplement: S5 Table — (DOCX) [file pone.0117261.s015.docx]

**S5 Table.** Top ten up-regulated *ERG*s (FC, FDR *q*-value < 0.05).

| ***ERG*s** | **ID**^A^ | ***E. coli* response** | **Combined inh.** | **CD14 inh.** | **C3 inh.** | **Biological process**^B^ |
| --- | --- | --- | --- | --- | --- | --- |
| **IL-6** | 8131803 | **129.9** | -10.7 | -2.8 | 1.2 | Acute phase response |
| **IRG1**^C^ | 7969482 | **84.4** | -13.1 | -4.1 | 1.4 | Propionate catabolic process |
| **MIR155HG**^D^ | 8068022 | **29.4** | -6.0 | -2.3 | 1.3 | MI0000681^E,F^ |
| **IL-12B** | 8115570 | **28.2** | -6.7 | -4.0 | 2.1 | T-helper 1 type immune response |
| **CCL20** | 8048864 | **23.3** | -4.8 | -1.6 | *n.s.*^G^ | Cell-cell signaling; chemotaxis |
| **IL-1A** | 8054712 | **22.1** | -2.9 | -1.6 | *n.s.* | Anti-apoptosis |
| **CXCL10** | 8101126 | **19.6** | -3.2 | -5.4 | 2.6 | Cell-cell signaling; chemotaxis |
| **IFIT1**^H^ | 7929065 | **17.6** | -6.6 | -7.0 | 1.7 | Negative regulation of defense response to virus by host^I^ |
| **IFIT2** | 7929047 | **15.4** | -4.3 | -3.6 | 1.6 | Response to virus^I^ |
| **IFIT3** | 7929052 | **15.2** | -4.7 | -3.7 | 1.5 | Cellular response to interferon-alpha^I^ |

^A^ Affymetrix transcript ID; see S7 Table for data from a C5-deficient patient

^B^ Gene ontology (GO_BP) annotations were retrieved from UniProtKB-GOA.

^C^ IRG1, immune-responsive 1 homolog

^D^ MIR155HG, non-protein coding host gene of microRNA155 (miR155)

^E^ microRNA Accession number

^F^ miR155 targets (according to IPA): AICDA, BCL2, CD47, CD69, CEBPB, DUSP5, FADD, IKBKE, Ikk (family), IL13RA1, INPP5D, JARID2, MAF, RIPK1, SOCS, SPI1, TAB2

^G^ *n.s.*, not significant

^H^ IFIT1, interferon-induced protein with tetratricopeptide repeats 1

^I^ Type I interferon-mediated signaling pathway
