# Supplementary material for: CD14 and Complement Crosstalk and Largely Mediate the Transcriptional Response to Escherichia coli in Human Whole Blood as Revealed by DNA Microarray
Source: PLoS One. 2015 Feb 23;10(2):e0117261. doi: 10.1371/journal.pone.0117261 (PMC4338229; doi:10.1371/journal.pone.0117261)
Supplement: S7 Table — (DOCX) [file pone.0117261.s017.docx]

**S7 Table.** Top ten up-regulated *ERG*s and their responses upon C5-deficiency (FC, FDR *q*-value < 0.05).

| ***ERG*s** | **ID**^A^ | ***E. coli response*** | **Combined inh.** | **CD14 inh.** | **C3 inh.** | **Biological process**^B^ |
| --- | --- | --- | --- | --- | --- | --- |
| **IL-6** | 8131803 | **153.2** | -44.40 | -20.57 | *n.s.*^C^ | Acute phase response |
| **IRG1**^D^ | 7969482 | **91.67** | -61.60 | -35.20 | *n.s.* | Propionate catabolic process |
| **MIR155HG**^E^ | 8068022 | **33.98** | -19.20 | -8.57 | *n.s.* | MI0000681^F,G^ |
| **IL-12B** | 8115570 | **52.43** | -34.72 | -16.18 | *n.s.* | T-helper 1 type immune response |
| **CCL20** | 8048864 | **20.86** | -12.18 | -8.67 | *n.s.* | Cell-cell signaling; chemotaxis |
| **IL-1A** | 8054712 | **14.91** | -5.06 | -3.73 | *n.s.* | Anti-apoptosis |
| **CXCL10** | 8101126 | **19.60** | -12.21 | -12.67 | 1.62 | Cell-cell signaling; chemotaxis |
| **IFIT1**^H^ | 7929065 | **36.52** | -16.84 | -17.33 | *n.s.* | Negative regulation of defense response to virus by host^I^ |
| **IFIT2** | 7929047 | **16.77** | -8.68 | -8.34 | *n.s.* | Response to virus^I^ |
| **IFIT3** | 7929052 | **19.11** | -10.47 | -7.32 | *n.s.* | Cellular response to interferon-alpha^I^ |

^A^ Affymetrix transcript ID

^B^ Gene ontology (GO_BP) annotations were retrieved from UniProtKB-GOA.

^C^ *n.s.*, not significant

^D^ IRG1, immune-responsive 1 homolog

^E^ MIR155HG, non-protein coding host gene of microRNA 155 (miR155)

^F^ microRNA Accession number

^G^ miR155 targets (according to IPA): AICDA, BCL2, CD47, CD69, CEBPB, DUSP5, FADD, IKBKE, Ikk (family), IL13RA1, INPP5D, JARID2, MAF, RIPK1, SOCS, SPI1, TAB2

^H^ IFIT1, interferon-induced protein with tetratricopeptide repeats 1

^I^ Type I interferon-mediated signaling pathway
