# Supplementary material for: CD14 and Complement Crosstalk and Largely Mediate the Transcriptional Response to Escherichia coli in Human Whole Blood as Revealed by DNA Microarray
Source: PLoS One. 2015 Feb 23;10(2):e0117261. doi: 10.1371/journal.pone.0117261 (PMC4338229; doi:10.1371/journal.pone.0117261)
Supplement: S8 Table — (DOCX) [file pone.0117261.s018.docx]

**S8 Table**. Top ten down-regulated *ERG*s and their responses upon C5-deficiency (FC, FDR *q*-value < 0.05).

| ***ERG*s** | **ID**^A^ |  | ***E. coli*** | **Combined inh.** | **CD14 inh.** | **C3 inh.** | **Biological process**^B^ |
| --- | --- | --- | --- | --- | --- | --- | --- |
| **MERTK**^C^ | 8044391 |  | **-1.3** | 1.3 | *n.s.*^D^ | *n.s.* | Positive regulation of phagocytosis |
| **VCAN**^E^ | 8106743 |  | **-3.8** | 4.5 | 3.4 | *n.s.* | Cell adhesion |
| **miR223** | 8167971 |  | **-1.7** | 3.3 | 1.6 | 1.8 | MI0000300^F,G^ |
| **CSF1R**^H^ | 8115076 |  | **-4.5** | 5.2 | 3.3 | *n.s.* | Cell proliferation |
| **CD14** | 8114612 |  | **-1.9** | 3.1 | 1.9 | 1.3 | Toll signaling pathway |
| **THBD**^I^ | 8065353 |  | **-1.1** | 2.0 | *n.s.* | 1.6 | Blood coagulation |
| **CD163** | 7960794 |  | **-1.5** | 2.1 | *n.s.* | *n.s.* | Acute phase response |
| **FOS**^J^ | 7975779 |  | **-1.8** | 4.9 | 1.5 | 1.3 | Toll signaling pathway |
| **NLRP12**^K^ | 8039096 |  | **-3.0** | 4.4 | 1.9 | *n.s.* | Negative regulation of Toll signaling pathway; positive regulation of IL-1b secretion |
| **FGL2**^L^ | 8140463 |  | **-2.0** | 2.6 | 1.5 | *n.s.* | Signal transduction |

^A^ Affymetrix transcript ID

^B^ Gene ontology (GO_BP) annotations were retrieved from UniProtKB-GOA.

^C^ C-mer proto-oncogene tyrosine kinase

^D^ *n.s.*, not significant

^E^ Versican

^F^ microRNA Accession number

^G^ miR223 (according to IPA): targets E2F1 and MEF2C

^H^ Colony stimulating factor 1 receptor

^I^ Thrombomodulin

^J^ FBJ murine osteosarcoma viral oncogene homolog

^K^ NLR family, pyrin domain containing 12

^L^ Fibrinogen-like 2
