# Supplementary material for: CD14 and Complement Crosstalk and Largely Mediate the Transcriptional Response to Escherichia coli in Human Whole Blood as Revealed by DNA Microarray
Source: PLoS One. 2015 Feb 23;10(2):e0117261. doi: 10.1371/journal.pone.0117261 (PMC4338229; doi:10.1371/journal.pone.0117261)
Supplement: S9 Table — (DOCX) [file pone.0117261.s019.docx]

**S9 Table.** Gene annotation enrichment analysis of specific subsets of C3- and/or C5aR-dependent *ERGs* (DAVID^A^; *p*<0.05).

| **Category /** *Subcategory* | **n**^B^ | **Molecular pathway (KEGG)** | **Transcription factor** |
| --- | --- | --- | --- |
| **C3-DG**^C^ |  |  |  |
| *Reversible C5aR-independent* | 251 | Hematopoietic cell lineage, Cytokine-cytokine receptor interaction, Chemokine signaling pathway | STAT5B, STAT3, AML1 |
| *Augmentable C5aR-independent* | 415 | RIG-I-like receptor signaling pathway, Cytosolic DNA-sensing pathway, Toll-like receptor signaling pathway | IRF2, IRF1, OCT |
| **C5aR-DG**^D^ | 249 | Cytokine-cytokine receptor interaction, Toll-like receptor signaling pathway,  NOD-like receptor signaling pathway | IRF2, CEBPB, AP1 |
| *Reversible* | 140 | Cytokine-cytokine receptor interaction, NOD-like receptor signaling pathway, MAPK signaling pathway | AP1, NFKAPPAB65, BACH1 |
| *Reversible C3-independent* | 57 | *n.s.*^E^ | SOX5, MEF2, HTF |
| *Reversible C3-dependent* | 83 | Cytokine-cytokine receptor interaction, ErbB signaling pathway, Bladder cancer | NFKAPPAB65 |
| *Augmentable* | 109 | Toll-like receptor signaling pathway, Cytokine-cytokine receptor interaction,  NOD-like receptor signaling pathway | CEBPB, IRF2, FREAC7 |
| *Augmentable C3-dependent* | 78 | Toll-like receptor signaling pathway, Cytosolic DNA-sensing pathway, NOD-like receptor signaling pathway | FREAC7, CEBPB, HLF |

^A^ According to DAVID Bioinformatics Resources 6.7 (<http://david.abcc.ncifcrf.gov:8080/>)

^B^ n, number of genes

^C^ C3-dependent genes (sensitive to inhibition of C3 with compstatin)

^D^ C5aR-dependent genes (sensitive to inhibition of C5a receptor 1 (CD88) with C5aR antagonist)

^E^ *n.s.*, no significant hit
