# Supplementary material for: CD14 and Complement Crosstalk and Largely Mediate the Transcriptional Response to Escherichia coli in Human Whole Blood as Revealed by DNA Microarray
Source: PLoS One. 2015 Feb 23;10(2):e0117261. doi: 10.1371/journal.pone.0117261 (PMC4338229; doi:10.1371/journal.pone.0117261)
Supplement: S10 Table — (DOCX) [file pone.0117261.s020.docx]

**S10 Table.** Clusters of IAE genes, associated types of crosstalk and functional annotations (DAVID^A^, *p*<0.1).

| **Cluster** | **Numbers of *ERG*s** | |  | **IAE-I** | | **Transcription**  **factor (n)** | **Biological process (n)** | **Subcellular**  **compartment (n)** |
| --- | --- | --- | --- | --- | --- | --- | --- | --- |
|  | **total** | **FC > 2** |  | **n**^B^ | **Type of crosstalk (n)** |  |  |  |
| **1** | 62  (*down*^C^) | 3 |  | 46 | Synergy (34)  Counteraction I (9)  Neg. Redundancy (3) | PPARG (49) | Organic acid biosynthetic process (5)  Carboxylic acid biosynthetic process (5)  Icosanoid metabolic process (3) | Plasma membrane part (16)  Endoplasmic reticulum (9) |
| **2** | 46  (*down*) | 8 |  | 11 | Synergy (1)  Counteraction I (2)  Counteraction II (7)  Redundancy (1) | BACH2 (25)  NRF2 (19)  MYB (25) | Regulation of cellular component size (5)  Response to vitamin (3)  Regulation of cell growth (4) | Actin cytoskeleton (4)  Extracellular space (5) |
| **3** | 23  (*up*^C^) | 23 |  | 12 | Counteraction I (8)  Synergy (4) | NFKB (15)  NFKAPPAB (12)  NKX25 (15) | Regulation of smooth muscle cell proliferation (4)  Regulation of cytokine biosynthetic process (4)  Positive regulation of immune system process (5) | Extracellular space (8) |
| **4** | 48  (*up*) | 43 |  | 18 | Synergy (10)  Counteraction I (8) | FOXO1 (20)  STAT5B (16)  IRF1 (13) | Response to cytokine stimulus (4)  Response to progesterone stimulus (3)  Response to organic substance (8) | Cell surface (6)  Membrane raft (3) |
| **5** | 68  (*up*) | 7 |  | 20 | Synergy (9)  Counteraction I (9)  Counteraction III (1)  Redundancy (1) | AP1 (46)  MEF2 (51)  CEBPB (41) | Membrane protein proteolysis (3)  Response to lipopolysaccharide (4)  Negative regulation of multicellular organismal process (5) | Integral to  plasma membrane (11)  Basolateral  plasma membrane (5) |
| **6** | 4  (*up*) | 1 |  | 3 | Synergy (1)  Counteraction I (3) | *n.s.*^D^ | *n.s.* | *n.s.* |

^A^ According to DAVID Bioinformatics Resources 6.7 (http://david.abcc.ncifcrf.gov:8080/)

^B^ n, number of IAE-I genes or of IAE genes associated with gene annotations (transcription factor binding sites, biological process, subcellular compartment)

^C^ *U*p- or down-regulation in response to *E. coli*

^D^ *n.s.*, no significant hit
