# Supplementary material for: CD14 and Complement Crosstalk and Largely Mediate the Transcriptional Response to Escherichia coli in Human Whole Blood as Revealed by DNA Microarray
Source: PLoS One. 2015 Feb 23;10(2):e0117261. doi: 10.1371/journal.pone.0117261 (PMC4338229; doi:10.1371/journal.pone.0117261)
Supplement: S11 Table — (DOCX) [file pone.0117261.s021.docx]

**S11 Table.** *ERG*s of the IPA canonical pathway *Role of Pattern Recognition Receptors in Recognition of Bacteria and Viruses* - Expression data from a C5-deficient background.

| ***ERG*s** | **Transcript**  **ID** | ***E. coli*** ^A^ | **healthy vs. C5D**^B^ | **Combined inh.** | **CD14 inh.** | **C3 inh.** |
| --- | --- | --- | --- | --- | --- | --- |
| *Cytokines* |  |  |  |  |  |  |
| **IL-6** | 8131803 | **153.17** | *n.s.*^C^ | -44.40 | -20.57 | *n.s.* |
| **IL-12B** | 8115570 | **52.43** | -1.86 | -34.72 | -16.18 | *n.s.* |
| **TNF** | 8118142 | **7.95** | *n.s.* | -3.22 | -2.92 | *n.s.* |
| **IL-1B** | 8054722 | **2.19** | *n.s.* | -1.33 | -1.34 | *n.s.* |
| **IL-10** | 7923907 | **2.23** | *n.s.* | -2.07 | -1.90 | *n.s.* |
| **IFNB1** | 8160360 | **2.31** | *n.s.* | -2.22 | -1.99 | 1.69 |
| *Pattern recognition – extracellular* | | | | |  |  |
| **PTX3** | 8083594 | **28.00** | -4.22 | -10.05 | -7.85 | *n.s.* |
| **CFB** | 8179351 | **6.52** | -1.49 | -7.83 | -6.08 | *n.s.* |
| **C3** | 8033257 | **3.97** | *n.s.* | -2.82 | -2.62 | 1.38 |
| *Pattern recognition – plasma membrane* | | | | |  |  |
| **C5L2 (GPR77)** | 8029914 | **-2.28** | *n.s.* | 3.24 | *n.s.* | *n.s.* |
| **TLR1** | 8099834 | **-2.80** | *n.s.* | 3.50 | *n.s.* | *n.s.* |
| **CLEC7A (Dectin-1)** | 7961120 | -1.88 | *n.s.* | 2.80 | *n.s.* | *n.s.* |
| **C5AR1** | 8029907 | *n.s.* | -2.37 | 1.56 | *n.s.* | *n.s.* |
| **TLR6** | 8099841 | **-3.72** | 1.59 | 5.38 | 1.66 | *n.s.* |
| **CD14** | 8114612 | -1.86 | -2.51 | 3.07 | 1.88 | *n.s.* |
| *Pattern recognition - intracellular* | | | | |  |  |
| **IFIH1 (MDA-5)** | 8056285 | **5.45** | *n.s.* | -3.82 | -3.61 | *n.s.* |
| **OAS1** | 7958884 | **8.03** | -1.75 | -6.27 | -5.32 | *n.s.* |
| **DDX58 (RIG-1)** | 8160559 | **4.05** | *n.s.* | -3.17 | -3.94 | 1.36 |
| **OAS3** | 7958895 | **9.61** | -2.72 | -8.47 | -6.60 | *n.s.* |
| **OAS2** | 7958913 | **5.48** | -1.66 | -4.95 | -3.79 | *n.s.* |
| *Inflammasome* | |  |  |  |  |  |
| **NLRP3 (NALP3)** | 7911178 | **2.03** | 1.68 | -4.95 | -3.79 | *n.s.* |
| P2RX7 | 7959251 | **5.78** | -3.25 | -4.75 | -4.12 | -1.58 |
| **NLRC4 (IPAF)** | 8051396 | **-3.60** | *n.s.* | 4.63 | 2.09 | 1.64 |
| *Intracellular signal transduction* | | | | |  |  |
| **RIPK2 (RIP2)** | 8147206 | **2.36** | *n.s.* | -1.85 | -2.10 | *n.s.* |
| **EIF2AK2 (PKR)** | 8051501 | **4.46** | -1.72 | -3.91 | -3.57 | *n.s.* |
| *Transcriptional regulators* | |  |  |  |  |  |
| **NFKB1 (p105)** | 8096635 | **3.11** | *n.s.* | -2.67 | -1.96 | *n.s.* |
| **IRF7** | 7945462 | **2.92** | *n.s.* | -2.53 | -2.19 | *n.s.* |
| **NFKB2 (p49/p100)** | 7930074 | 1.80 | *n.s.* | -1.62 | -1.36 | *n.s.* |

^A^ *ERG*s with fold change (FC) expression in response to *E. coli* above two-fold are indicated in bold.

^B^ Fold change between *E. coli* responses in C5-deficient patient (C5D) compared to healthy control (Limma): Increased expression in C5D compared to control results in a negative value, decreased expression results in a positive value

^C^ *n.s.*, not significant with FDR *q*-value > 0.05 (Limma)
